# ACLY‐Driven Metabolic Reprogramming Promotes Histone Acetylation and Inflammation‐Associated Fibrosis in Chronic Kidney Disease

**DOI:** 10.1002/advs.75247

**Published:** 2026-04-16

**Authors:** Chunxiu Du, Dhanunjay Mukhi, Lingzhi Li, Chenyu Li, Siyu Pan, Bernhard Dumoulin, Eunji Ha, Lakshmi P Kolligundla, Yanjuan Hou, Jonathan Levinsohn, Chaelin Kang, Konstantin Adrian Klötzer, Junnan Wu, Samer Mohandes, Kathryn E Wellen, Katalin Susztak

**Affiliations:** ^1^ Institutes for Diabetes Obesity and Metabolism Department of Medicine Perelman School of Medicine University of Pennsylvania Philadelphia Pennsylvania USA; ^2^ Renal Electrolyte & Hypertension Division Department of Medicine Perelman School of Medicine University of Pennsylvania Philadelphia Pennsylvania USA; ^3^ Department of Genetics Perelman school of Medicine University of Pennsylvania Philadelphia Pennsylvania USA; ^4^ Abramson Family Cancer Research Institute Perelman School of Medicine University of Pennsylvania Philadelphia Pennsylvania USA

**Keywords:** acetyl‐CoA metabolism, chromatin accessibility, H3K27ac, kidney fibrosis

## Abstract

The mechanisms by which metabolic stress drives epigenetic dysregulation and fibrosis in chronic kidney disease (CKD) remain incompletely understood. Using quantitative histone proteomics in murine fibrosis models, we uncovered a selective increase in  histone H3 lysine 27 acetylation (H3K27ac) as a conserved epigenetic feature. Unbiased metabolomics revealed citrate accumulation, nominating ATP‐citrate lyase (ACLY) as a driver of acetyl‐CoA‐dependent histone acetylation. In murine models of folic acid and unilateral ureteral obstruction, ACLY expression, acetyl‐CoA levels, and H3K27ac were increased in injured kidneys. Tubule‐specific *Acly* deletion reduced acetyl‐CoA, H3K27ac, and attenuated tubulointerstitial fibrosis. Chromatin accessibility profiling revealed that loss of *Acly* decreased accessibility at pro‐inflammatory loci, including *Jak1* and *Jak2*, with reduced transcriptional output. These transcriptional and epigenetic signatures were observed in human CKD samples, where higher ACLY expression correlated with worse kidney function and increased JAK1/2 expression. Notably, ACLY inhibitors, including bempedoic acid and BMS‐303141 recapitulated the antifibrotic effects of *Acly* deletion in vivo in mice, supporting the therapeutic repurposing of ACLY inhibitors for CKD. Together, our findings position ACLY as a key metabolic‐epigenetic checkpoint of kidney fibrosis and a promising, druggable target for halting CKD progression.

## Graphical abstract

This study reveals a citrate–ACLY–H3K27ac metabolic–epigenetic axis driving inflammatory gene activation and kidney fibrosis, highlighting ACLY inhibition as a potential therapeutic strategy for chronic kidney disease (CKD).

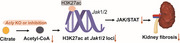

## 1 Introduction

Chronic kidney disease (CKD) affects over 800 million individuals worldwide, posing a major global health burden [1]. Despite improvements in detection and disease management, CKD remains a life‐threatening condition, as many patients develop cardiovascular complications or die prematurely before reaching end‐stage kidney disease (ESKD) [2]. Diabetes is the leading cause of CKD and ESKD, accounting for nearly half of all cases. In addition, recent evidence highlights that acute kidney injury (AKI)‐triggered by transient ischemia or nephrotoxins‐can drive CKD progression through persistent tubular injury. Episodes of poor glycemic control in diabetes may similarly induce a “metabolic memory” effect that continues to promote kidney damage, even after normalization of blood glucose levels   [3].

The underlying molecular mechanisms sustaining these injury states remain unclear. Epigenetic reprogramming is a widely proposed explanation, but definitive evidence in human kidney tissue is lacking. The epigenome, through modifications such as histone acetylation and DNA methylation, governs chromatin accessibility and transcriptional activity [4, 5]. Because these modifications are propagated during cell division, they offer a plausible mechanism for long‐term transcriptional changes that drive disease. Epigenome‐wide association studies (EWAS) in humans have revealed widespread epigenetic variation that correlates with kidney function traits, including estimated glomerular filtration rate (eGFR) and albuminuria [4, 5, 6]. In addition, altered epigenetic changes have been reported in peripheral blood from CKD patients [7]. However, a mechanistic link between specific epigenetic changes and kidney fibrosis in human tissue remains to be established.

Cellular metabolism may represent a key upstream regulator of the epigenome. Chromatin‐modifying enzymes require metabolites as cofactors or substrates: for example, S‐adenosylmethionine donates methyl groups for DNA and histone methylation, while acetyl‐CoA donates acetyl groups for histone acetylation [8, 9, 10, 11, 12]. Thus, fluctuations in metabolite availability can directly alter chromatin structure and transcriptional programs. This connection could be particularly relevant in renal tubular epithelial cells, which have high metabolic demands, depend on fatty acid oxidation, and are highly sensitive to metabolic stress [13, 14].

Histone acetylation, particularly on histone H3, represents a major epigenetic mechanism associated with open chromatin and active transcription. In the nucleus, acetyl‐CoA is primarily generated from citrate via ATP‐citrate lyase (ACLY) [15], and from acetate via acyl‐CoA synthetase short‐chain family member 2 (ACSS2) [12, 16]. Previous work from our group showed that under metabolic stress, ACSS2 in the kidney primarily supports lipid synthesis rather than histone acetylation [17]. By contrast, ACLY‐derived acetyl‐CoA links carbohydrate and lipid metabolism with histone acetylation and gene regulation.

Previous work has implicated ACLY in fibrotic remodeling in other organs, including the heart and vasculature, where ACLY‐dependent acetyl‐CoA production promotes H3K27 acetylation and myofibroblast activation [18, 19, 20]. However, the metabolic–epigenetic mechanisms that sustain fibrotic transcriptional programs in the kidney, and their relevance in human CKD tissue, remain poorly defined.

Mendelian randomization studies suggest that genetically reduced ACLY expression may protect against CKD, though these findings are based on blood expression quantitative trait loci (eQTLs), not kidney tissue, and do not extend to continuous kidney function traits [21]. Preclinical studies have linked ACLY to extracellular matrix production and fibrosis, but kidney‐specific epigenetic consequences and long‐term functional outcomes remain poorly characterized [18, 20].

Here, we address these knowledge gaps directly. We perform integrative analysis of human kidney biopsies and preclinical models using quantitative histone proteomics and untargeted metabolomics, complemented by chromatin accessibility profiling. We define a role for citrate‐ACLY‐H3K27ac‐JAK axis in renal tubular cells in kidney disease and fibrosis.

## 2 Results

### 2.1 Unbiased Histone Profiling Identifies Higher H3K27 Acetylation in Mouse Models of Kidney Fibrosis

While epigenetic dysregulation has been proposed in CKD, a systematic analysis of histone modifications has not been performed in kidney fibrosis. In humans, the four core histone proteins are subject to diverse post‐translational modifications (PTMs) at their N‐terminal tails, including acetylation (ac), methylation (me), ubiquitination, crotonylation, and phosphorylation. These modifications regulate chromatin accessibility and transcription, and accumulating evidence suggests they contribute to CKD pathogenesis.

**FIGURE 1:**
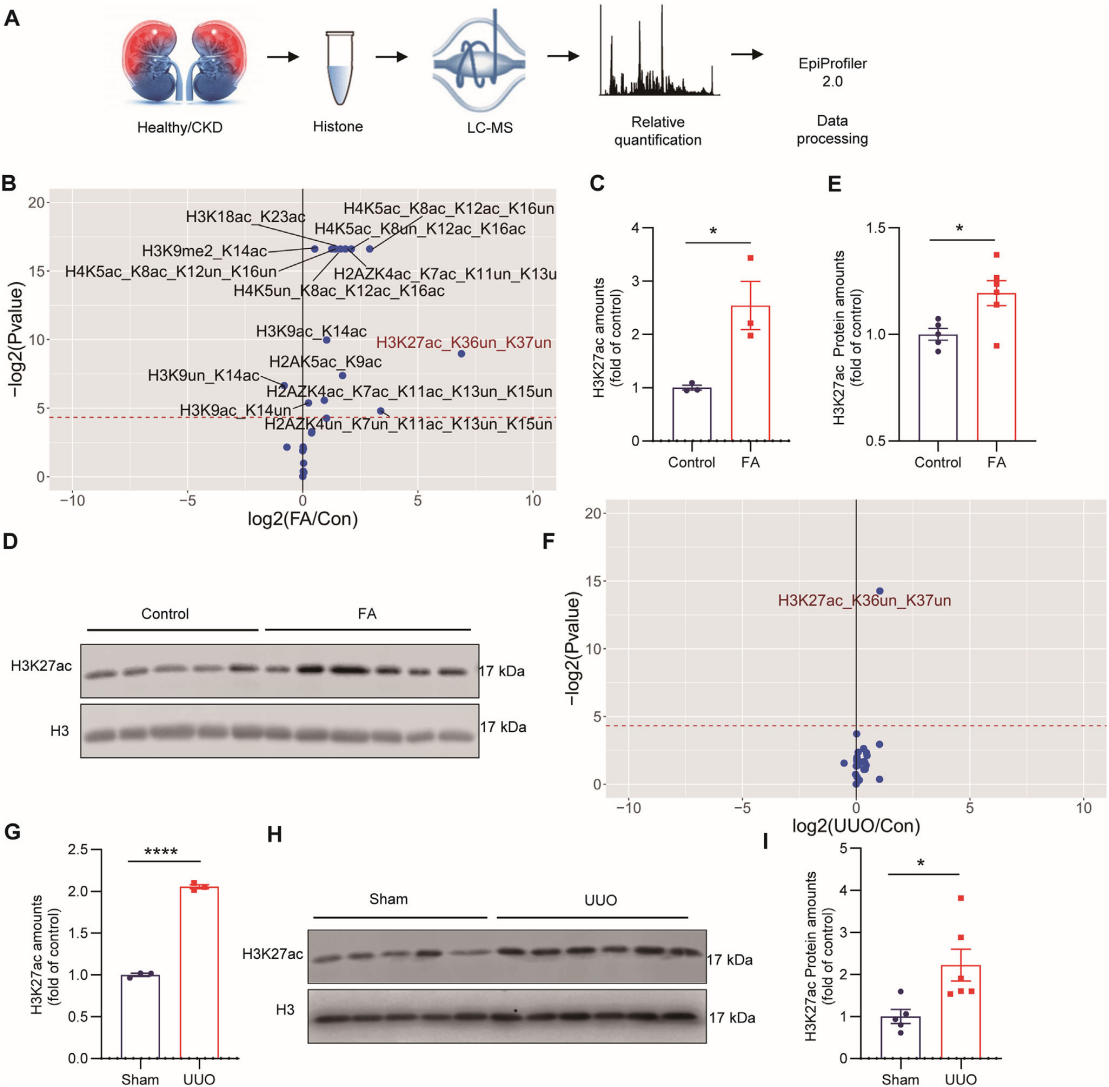
Unbiased histone profiling identifies higher H3K27 acetylation levels in mouse models of kidney fibrosis. (A) Schematic of workflow for histone modification profiling using LC‐MS/MS and EpiProfiler 2.0 in kidneys from healthy and diseased mice. (B,C) Volcano plot (log2 fold change vs. –log2(*p*‐value))  of histone modifications comparing control and folic acid (FA)–treated kidneys (B), and quantification of histone H3 lysine 27 acetylation (H3K27ac) levels in FA and control kidneys measured by LC‐MS/MS (C). ^*^ *p* < 0.05, n = 3. (D) Representative western blots of H3K27ac and total H3 in nuclei isolated from FA and control kidney samples. (E) Quantification of H3K27ac protein levels in nuclei isolated from FA and control kidneys. ^*^ *p* < 0.05, n = 5–6. (F,G) Volcano plot (log2 fold change vs. –log2(*p*‐value)) of histone modifications in sham and unilateral ureteral obstruction (UUO) kidneys (F), and quantification of H3K27ac levels in UUO and sham kidneys measured by LC‐MS/MS (G). ^****^ *p* < 0.0001, n = 3. (H) Representative western blots of H3K27ac and total H3 in nuclei isolated from UUO and sham kidney samples. (I) Quantification of H3K27ac protein levels in nuclei isolated from UUO and sham kidney samples. ^*^ *p* < 0.05, n = 5–6. Data represent mean ± SEM. Unpaired two‐tailed Student's *t* test for C, E, G, I.

To systematically define histone PTM changes in fibrosis, we performed unbiased histone proteomic profiling using LC‐MS/MS in two established murine models: folic acid (FA)–induced nephropathy, which causes direct proximal tubular injury, and unilateral ureteral obstruction (UUO), which produces obstruction‐driven tubular damage that ultimately affects both distal and proximal segments (Figure 1A). Following nuclear isolation and controlled digestion, mass spectrometry revealed widespread alterations in histone marks in fibrotic kidneys. In the FA model, the combinatorial modification H3K27ac_K36un_K37un emerged as the most consistently increased (Figure 1B,C). Consistently, western blot confirmed that H3K27ac protein abundance was markedly elevated in FA kidneys compared with controls (Figure 1D,E). We next examined the UUO model, where LC‐MS/MS similarly identified H3K27ac_K36un_K37un as the consistently increased modification (Figure 1F,G). This finding was further validated by western blot, which showed a robust increase in H3K27ac protein abundance in UUO kidneys (Figure 1H,I). Together, these results identify elevated H3K27ac as a conserved epigenetic hallmark of kidney fibrosis across distinct injury models.

### 2.2 Unbiased Metabolomics Analysis Identifies Higher Citrate Levels and ACLY Activity in Fibrotic Mouse Kidneys

**FIGURE 2:**
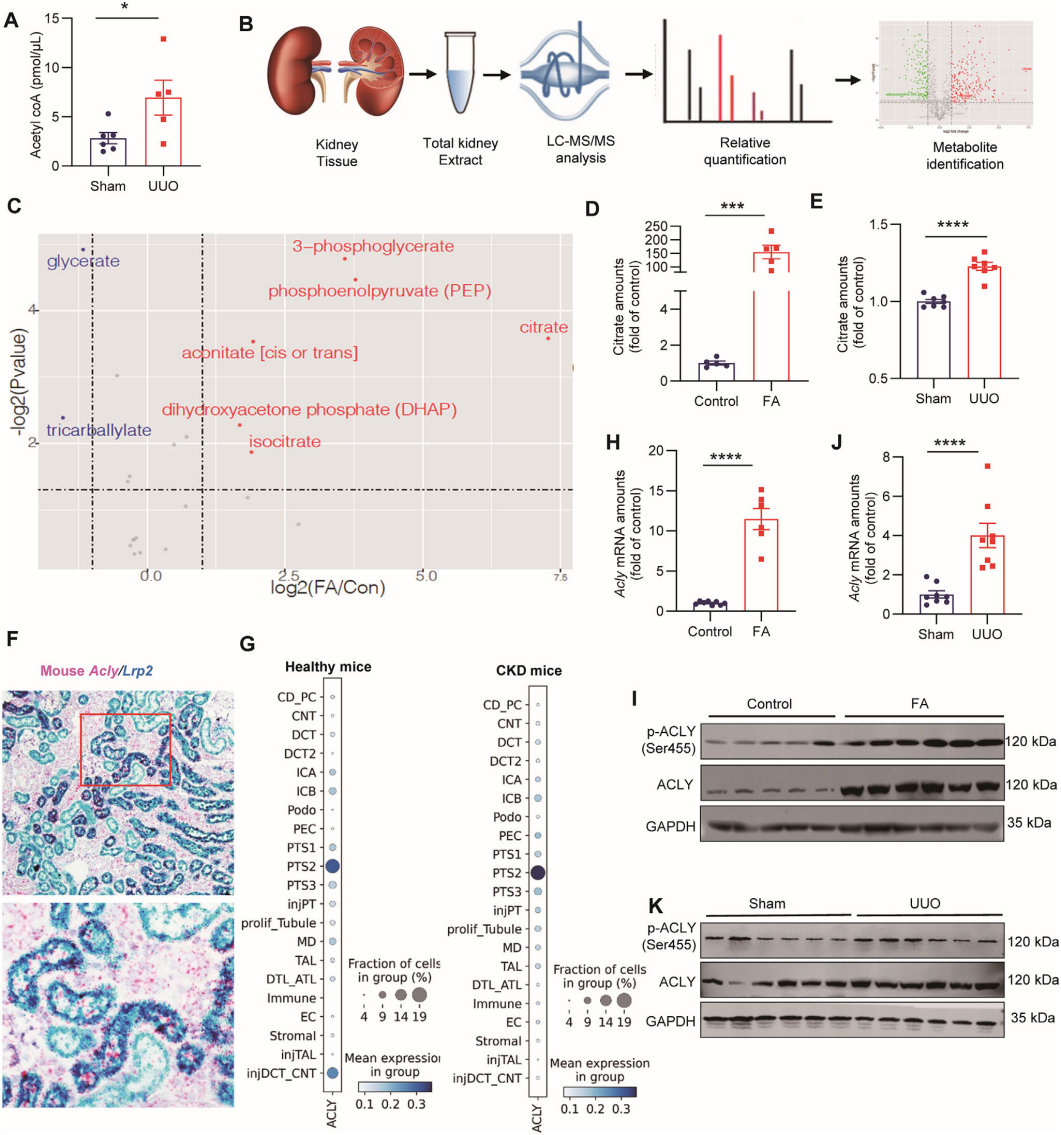
Unbiased metabolomics analysis identifies higher citrate levels and ACLY activity in fibrotic mouse kidneys. (A) Measurement of acetyl‐CoA levels in unilateral ureteral obstruction (UUO) kidneys vs. controls. Acetyl‐CoA levels were increased in the UUO group compared with controls. ^*^ *p* < 0.05, n = 5–6. (B) Schematic of an untargeted metabolomics workflow using LC‐MS/MS to analyze total kidney extracts from healthy and chronic kidney disease (CKD) mice. (C) Volcano plot (log2 fold change vs. –log2(*p*‐value)) showing altered metabolites in folic acid (FA)‐treated kidneys compared to controls. (D) Citrate levels were quantified by LC–MS/MS. Data are presented as fold change relative to the control group (control = 1.0). ^***^ *p* < 0.001, n = 5. (E) Citrate levels in UUO and control kidneys were measured using a citrate assay kit according to the manufacturer's instructions (Abcam, ab83396). Data are presented as fold change relative to the control group (normalized to 1.0). ^****^ *p* < 0.001, n = 7. (F) RNA in situ hybridization showing *Acly* (magenta) and *Lrp2* (blue) colocalization in renal tubules of mice; the down panel shows a magnified region. (G) Single‐cell RNA‐seq analysis showing *Acly* expression distribution across nephron segments in healthy and CKD mice (including UUO, folic acid nephropathy, adriamycin nephropathy, and diabetic kidney disease models). Expression is enriched in proximal tubule segments and in injured tubules across CKD models. CD_PC: Collecting Duct Principal Cells, CNT: Connecting Tubule Cells, DCT: Distal Convoluted Tubule Cells, DCT2: Distal Convoluted Tubule Segment 2, ICA: Intercalated Cells, Type A, ICB: Intercalated Cells, Type B, Podo: Podocytes, PEC: Parietal Epithelial Cells, PTS1: Proximal Tubule Segment 1, PTS2: Proximal Tubule Segment 2, PTS3: Proximal Tubule Segment 3, injPT: Injured Proximal Tubule Cells, prolif_Tubule: Proliferating Tubular Cells, MD: Macula Densa, TAL: Thick Ascending Limb, DTL_ATL: Descending Thin Limb/Ascending Thin Limb, Immune: Immune Cell Populations, EC: Endothelial Cells, Stromal: Stromal/Fibroblast Cells, injTAL: Injured Thick Ascending Limb, injDCT_CNT: Injured Distal Convoluted Tubule/Connecting Tubule Cells. (H,I) The expression of *Acly* mRNA (H) and protein levels (I) in FA‐ treated kidneys relative to controls. ^****^ *p* < 0.0001, n = 6–8. (J,K) The expression of *Acly* mRNA (J), ACLY and phospho ACLY protein levels (K) in UUO and Sham kidneys. ^****^ *p* < 0.0001, n = 8. Data represent mean ± SEM. Unpaired two‐tailed Student's *t* test for A, D, E, H, J.

To investigate whether metabolic alterations contribute to epigenetic changes in kidney fibrosis, we characterized metabolic shifts across complementary injury models. Because acetyl‐CoA is the essential donor for histone acetylation, we first performed targeted quantification of acetyl‐CoA in the UUO model and observed a clear increase compared with sham controls (Figure 2A). To define upstream pathways that may supply acetyl‐CoA, we next performed untargeted metabolomic profiling in the FA model (Figure 2B), which revealed broad accumulation of tricarboxylic acid (TCA) cycle intermediates, with citrate being among the most prominently elevated metabolites (Figure 2C,D). Finally, targeted citrate quantification confirmed that citrate accumulation also occurs in UUO kidneys (Figure 2E), indicating that increased citrate availability is a conserved metabolic feature of fibrotic kidneys. Since ACLY catalyzes the conversion of citrate to acetyl‐CoA, this suggests that ACLY activity may contribute to acetyl‐CoA availability and subsequent histone acetylation in fibrotic kidneys. RNA in situ hybridization demonstrated that *Acly* transcripts co‐localize with megalin (*Lrp2*), a marker of PT in mouse kidneys, confirming *Acly* expression in PT epithelial cells (Figure 2F). Consistent with this spatial localization, single‐cell RNA sequencing showed that *Acly* is enriched in PT segments and further increased in PT cells in mouse CKD. (Figure 2G). Importantly, both mRNA and protein levels of ACLY were markedly elevated in kidneys from FA‐treated (Figure 2H,I; Figure S1A,B) and UUO mice (Figure 2J,K; Figure S1C,D) compared to controls, indicating transcriptional and translational upregulation of ACLY in fibrotic kidneys. These results implicate ACLY as a key metabolic‐epigenetic integrator linking citrate metabolism to histone acetylation in kidney fibrosis.

### 2.3 Tubule‐Specific Deletion of *Acly* Attenuates Renal Fibrosis in UUO and FA Mouse Models by Reducing H3K27ac

**FIGURE 3:**
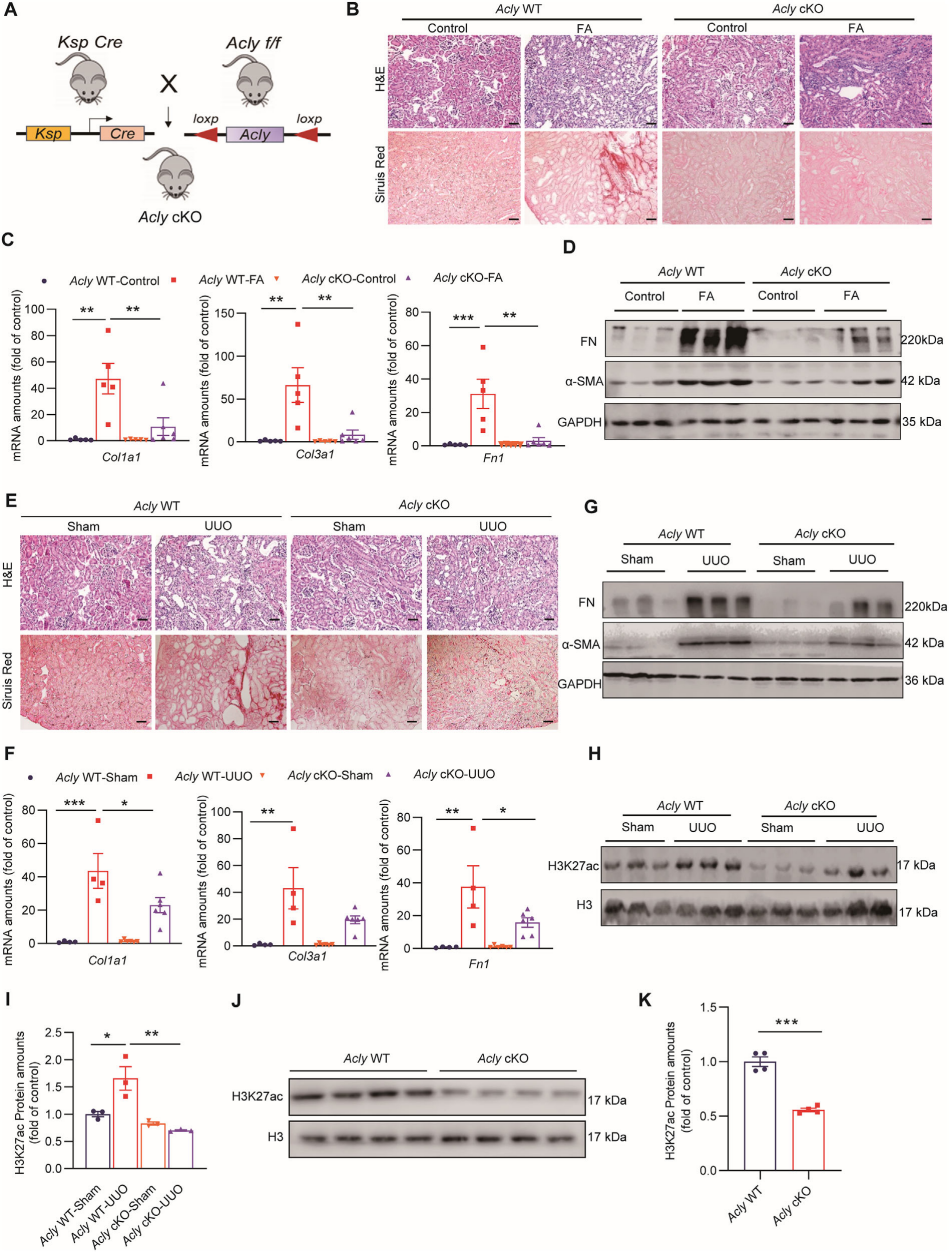
Tubule‐specific deletion of *Acly* attenuates kidney fibrosis in UUO and FA mouse models by reducing H3K27ac. (A) Generation of tubule‐specific *Acly* knockout mice (*Acly* cKO) by crossing *Ksp‐Cre* mice with *Acly* ^f/f^ mice. (B) Representative H&E and Sirius Red staining of kidney sections from WT and *Acly* cKO mice treated with vehicle or folic acid (FA). Scale bars, 50 µm. (C) Relative mRNA levels of fibrotic gene expression (*Col1a1*, *Col3a1*, and *Fn1*) in kidneys from *Acly* WT and *Acly* cKO mice treated with vehicle or FA, fibrotic gene expression was reduced in *Acly* cKO mice treated with FA compared with WT mice treated with FA. ^**^ *p* < 0.01, ^***^ *p* < 0.001, n=5–6. (D) Western blots analysis of fibronectin (FN) and α‐SMA protein levels in WT and *Acly* cKO mice kidneys treated with vehicle or FA. (E) Representative images of H&E (top) and Sirius Red (bottom) staining in kidneys from WT and *Acly* cKO mice after UUO or sham. Scale bars, 50 µm. (F) Relative mRNA levels of fibrotic genes *(Col1a1, Col3a1, and Fn1*) in kidneys from WT and *Acly* cKO mice after unilateral ureteral obstruction (UUO) or sham, fibrotic gene expression was reduced in *Acly* cKO UUO mice compared with WT UUO mice. ^*^ *p* < 0.05, ^**^ *p* < 0.01, ^***^ *p* < 0.001, n = 4–6. (G) Representative western blots of FN and α‐SMA in kidney lysates from WT and *Acly* cKO mice after UUO or sham. (H) Western blots showing histone H3 lysine 27 acetylation (H3K27ac) levels in WT and *Acly* cKO kidneys after UUO or sham. (I) Quantification of H3K27ac in (H), normalized to total H3. ^*^ *p* <0.05, ^**^ *p* < 0.01, n = 3. (J) Western blots showing H3K27ac levels decreased in *Acly* cKO primary tubule cells compared with WT cells. (K) Quantification of H3K27ac in (J), normalized to total H3. n = 4. Data represent mean ± SEM. One‐way ANOVA with Tukey's multiple comparisons test for C, F, I. Unpaired two‐tailed Student's *t* test for K.

To investigate the functional role of ACLY in kidney fibrosis, we generated a mouse model with tubule‐specific *Acly* deficiency using a conventional Cre‐lox strategy (*Acly* ^f/f^ mice) [22] and cross these mice with *Ksp‐Cre* transgenic mice to achieve tubule‐specific deletion (*Acly* ^fl/fl;Ksp‐Cre^), hereafter referred to as *Acly* conditional knockout (*Acly* cKO) mice. (Figure 3A). Genotyping (Figure S2A), together with mRNA and protein analyses, confirmed efficient *Acly* depletion in renal tubular epithelial cells (Figure S2B,C).

We observed markedly lower tubular injury and collagen deposition in *Acly* cKO FA kidneys compared with WT FA controls, as demonstrated by H&E and Sirius Red staining (Figure 3B; Figure S3A). Consistently, *Acly* cKO FA kidneys exhibited markedly decreased mRNA levels of fibrotic genes (*Col1a1*, *Col3a1*, *Fn1*) (Figure 3C) and reduced protein abundance of fibronectin (FN) and α‐SMA (Figure 3D; Figure S3B,C). Similar protective effects were observed in UUO models (Figure 3E–G; Figure S3D–F), supporting a protective role of tubular *Acly* deletion against renal fibrosis. Because ACLY supplies acetyl‐CoA for histone acetylation, we next examined whether *Acly* deletion alters protein acetylation during kidney fibrosis. Using a pan‐acetyl‐lysine antibody, we found that acetylation signals were predominantly localized within the nucleus, with no appreciable increase in cytoplasmic staining in UUO kidneys (Figure S4A), indicating that acetylation remodeling primarily occurs at the nuclear level and is closely associated with chromatin‐related processes. Subcellular fractionation followed by immunoblotting further confirmed that global acetylation signals were markedly enriched in the nuclear fraction of WT UUO kidneys but substantially attenuated in *Acly* cKO UUO kidneys, whereas cytoplasmic acetylation remained unchanged across groups (Figure S4B). We next examined the specific histone acetylation mark H3K27ac. The UUO‐induced increase in H3K27ac observed in WT kidneys was markedly reduced in *Acly* cKO kidneys (Figure 3H,I). Consistent with these in vivo findings, primary tubular epithelial cells from *Acly* cKO mice also exhibited markedly reduced H3K27ac levels, confirming that ACLY is required to maintain histone acetylation in tubular epithelial cells (Figure 3J,K).

Given that acetate can also serve as an upstream carbon source for acetyl‐CoA via acyl‐CoA synthetase short‐chain family 2 (ACSS2), we next quantified acetate levels in both fibrotic kidney tissues and in circulation. Notably, acetate concentrations were significantly reduced in fibrotic kidneys and were also decreased in serum (Figure S5A,B), indicating diminished acetate availability both locally and systemically in this disease context. Consistent with this limitation in substrate availability, ACSS2 protein expression was reduced under UUO conditions and was not further altered in *Acly*‐deficient UUO kidneys (Figure S5C), suggesting that the acetate‐ACSS2 pathway may not play a major compensatory role in sustaining acetyl‐CoA production during fibrosis.

Together, these findings demonstrate that tubule‐specific deletion of *Acly* protects against kidney fibrosis by disrupting citrate‐acetyl‐CoA flux and attenuating histone H3K27ac, highlighting ACLY as a critical metabolic‐epigenetic regulator in CKD pathogenesis.

### 2.4 ACLY Modulates Chromatin Accessibility and Fibrosis‐Related Gene Programs in Mouse Models of CKD

To better understand the protective role of ACLY in kidney fibrosis, we first examined whether its known function in lipid metabolism contributes to disease progression. ACLY catalyzes the conversion of citrate to acetyl‐CoA, providing substrate for de novo lipogenesis and fatty acid metabolism. Because proximal tubule (PT) cells rely heavily on fatty acid oxidation for energy production‐and defects in lipid handling have been implicated in CKD progression‐we tested whether loss of ACLY alters lipid metabolic pathways. Quantitative PCR analysis of key metabolic genes (*Acox1*, *Acox2*, *Cpt1a*, *Cpt2*, *Ppara*, *Plin2*, *Acaca*, *Acacb*, *Scap*, *Srebf1a*, *Fasn*) revealed no differences between *Acly* cKO and WT kidneys (Figure S6).

These data indicate that loss of ACLY does not produce major changes in the expression of key lipid metabolism–related genes in this model. Because our earlier results demonstrated that ACLY regulates H3K27ac levels‐a mark of active enhancers and promoters‐we next examined whether ACLY influences chromatin accessibility at pro‐fibrotic loci. To test this, we first performed Assay for Transposase‐Accessible Chromatin using sequencing (ATAC‐seq), a technique that maps genome‐wide chromatin accessibility by using a hyperactive Tn5 transposase to insert sequencing adapters into open chromatin regions. This approach identifies regions of the genome that are accessible to transcription factors and other regulatory proteins. We conducted ATAC‐seq in kidneys from WT and *Acly* cKO mice, which lack ACLY specifically in kidney tubule cells (Figure 4A), and identified a total of 43 754 accessible chromatin peaks across both groups, representing open chromatin regions (Figure 4B). These peaks were enriched primarily in promoters and distal intergenic regions. Line plot analysis centered on transcription start sites (TSS) revealed that global promoter accessibility was largely preserved in *Acly* cKO kidneys (Figure 4C), suggesting that ACLY does not broadly affect TSS‐proximal accessibility. Motif enrichment analysis of shared peaks between WT and *Acly* cKO kidneys revealed strong enrichment for nuclear receptor binding motifs, including RARα, ERRα, and PPARα (Figure 4D), consistent with the core transcriptional program in proximal tubule cells.

FIGURE 4ACLY modulates chromatin accessibility and fibrosis‐related gene programs in mouse models of CKD. (A) Kidneys from WT (n = 5) and *Acly* cKO (n = 3) mice were collected, nuclei were isolated, and chromatin accessibility was profiled by ATAC‐seq. (B) Genomic distribution of ATAC‐seq peaks shared between *Acly* cKO and WT kidneys. (C) Global accessibility profile of ATAC‐seq peaks across *Acly* WT and *Acly* cKO samples shows similar overall chromatin landscapes. (D) Motif enrichment analysis of shared accessible chromatin regions in *Acly* WT and *Acly* cKO kidneys. HOMER analysis of genomic regions that remained accessible in both *Acly* WT and *Acly* cKO kidneys identified enriched transcription factor motifs, including nuclear receptor (NR) motifs (RARa, ERRa, COUP‐TFII, PPRAa), hepatocyte nuclear factor (HNF) motifs, and CTCF/Zf motifs. *P*‐values indicate the statistical significance of motif overrepresentation compared with background sequences. (E) Volcano plot (log2 fold change vs. –log10(FDR)) showing differentially accessible peaks between *Acly* cKO and WT kidneys. (F) Pie chart quantifying the proportion of differentially accessible regions (DARs) with higher (5.4%), lower (7.6%), or unchanged accessibility (87%). (G,H) Gene Ontology (G) and Kyoto Encyclopedia of Genes and Genomes (KEGG) pathway (H) enrichment analyses were performed for genes associated with lower DARs in *Acly* cKO compared with WT kidneys. Enriched pathways highlight fibrosis‐related biological processes, including actin cytoskeleton organization, cell adhesion, and PI3K–AKT and MAPK signaling. (I) Schematic illustration of pathway overlap. Pathways enriched from genes associated with decreased DARs in *Acly* KO mice were compared with genes up‐regulated in proximal tubules from chronic kidney disease (CKD) patients. Overlap is expressed as the percentage of pathways derived from decreased DAR‐associated genes. (J) Pie chart showing that 15.2% of total downregulated DAR‐associated genes in *Acly* cKO mice (compared with WT controls) are shared with upregulated DAR‐associated genes in proximal tubules from CKD patients (compared with healthy controls). (K) Schematic illustration showing enrichment analysis of *Acly* cKO‐associated lower DAR‐related genes or shared genes. These genes were mapped to the nCounter Human Fibrosis V2 Panel (NanoString Technologies, Seattle, WA), which contains 760 fibrosis‐related genes categorized into four biological modules: inflammation, proliferation, initiation, and modification. (L) Functional categorization of genes linked to lower DARs using the nCounter Human Fibrosis V2 Panel. The majority of DAR‐associated genes mapped to the inflammation module, followed by proliferation, initiation, and modification modules. (M) Functional categorization of the shared DAR‐associated genes identified in (J). These genes were mapped to the nCounter Human Fibrosis V2 Panel. Shared genes were most frequently categorized into the proliferation and inflammation modules. (N,O) Genome browser tracks showing ATAC‐seq profiles from five WT (red) and three *Acly*‐deficient (KO; black) kidneys at the *Jak1* (N) and *Jak2* (O) loci. Previously reported H3K27ac ChIP‐seq peaks (purple) mark putative enhancer and promoter regions (Wilflingseder et al., *Nat Commun* 2020). Chromatin accessibility at these H3K27ac‐marked regulatory elements was markedly reduced in *Acly*‐deficient kidneys, indicating diminished activation of *Jak1* and *Jak2* regulatory regions upon ACLY loss. (P–R) Chromatin immunoprecipitation followed by quantitative PCR (ChIP‐qPCR) was performed to assess H3K27ac enrichment at the *Jak1* (P), *Jak2* (Q), and *Rpl30* (R) promoter regions in tubular epithelial cells treated with TGF‐β (5 ng/mL, 24 h). H3K27ac enrichment at the *Jak1* and *Jak2* promoters was attenuated in *Acly*‐deficient cells compared with WT cells, whereas enrichment at the *Rpl30* promoter remained unchanged. ^*^ *p* < 0.05, n = 4. (S, T) Relative *Jak1* and *Jak2* mRNA amounts in WT and *Acly* cKO kidneys under sham or UUO conditions. *Jak1* and *Jak2* mRNA expression was lower in *Acly* cKO unilateral ureteral obstruction (UUO) mice compared with WT UUO mice. ^**^ *p* < 0.01, n = 6. (U) Western blot analysis of phosphorylated and total JAK1 and JAK2 in kidneys from WT and *Acly* KO mice after UUO or Sham. Data represent mean ± SEM. Unpaired two‐tailed Student's *t* test for P, Q, R. One‐way ANOVA with Tukey's multiple comparisons test for S, T.

**Figure:**
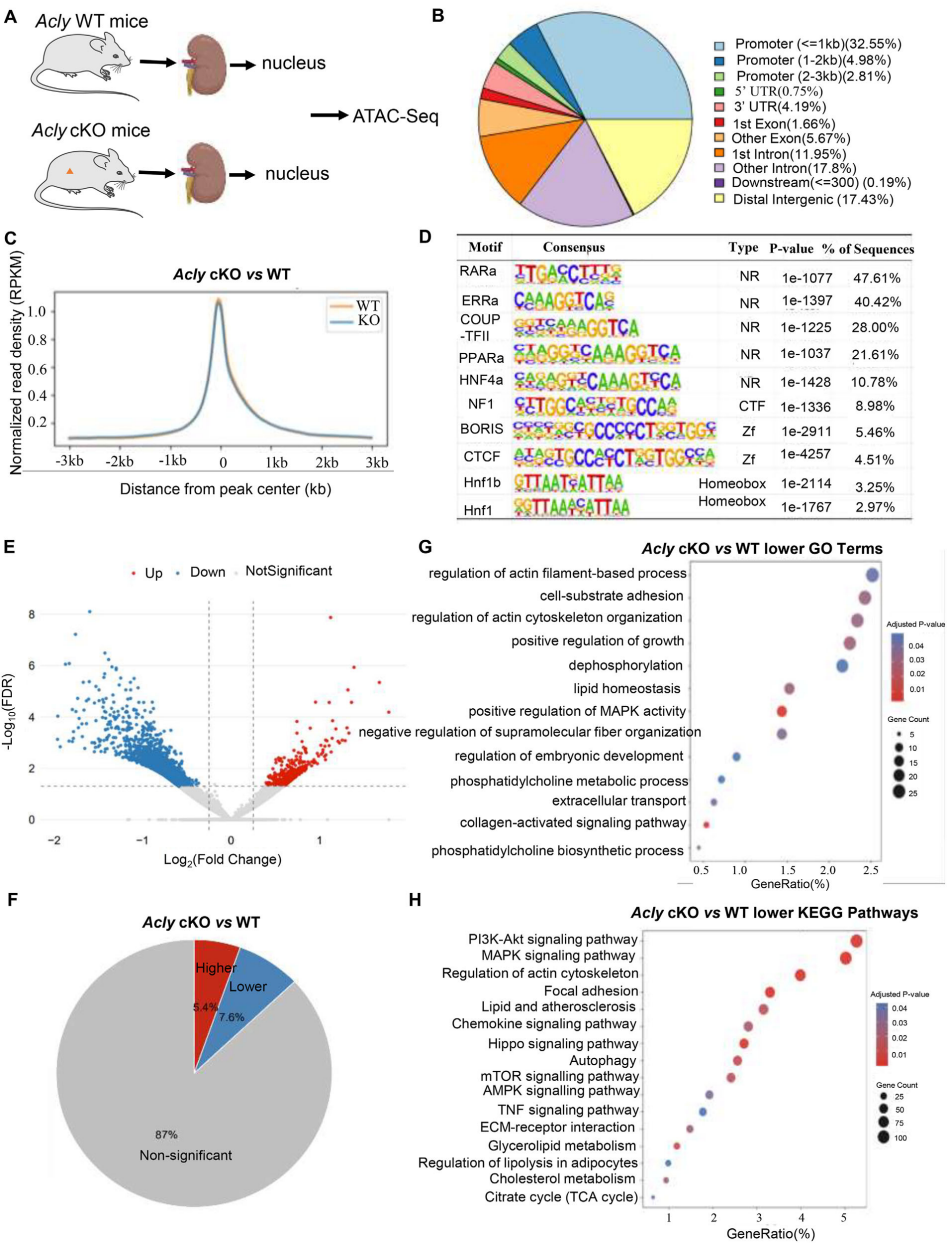

**Figure:**
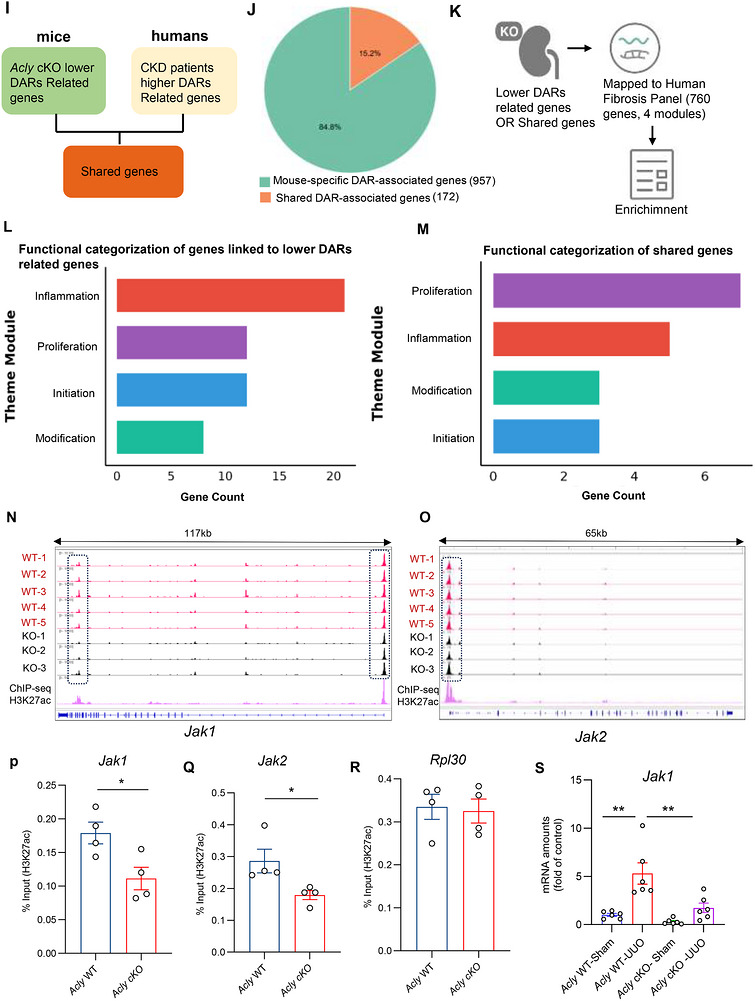

**Figure:**
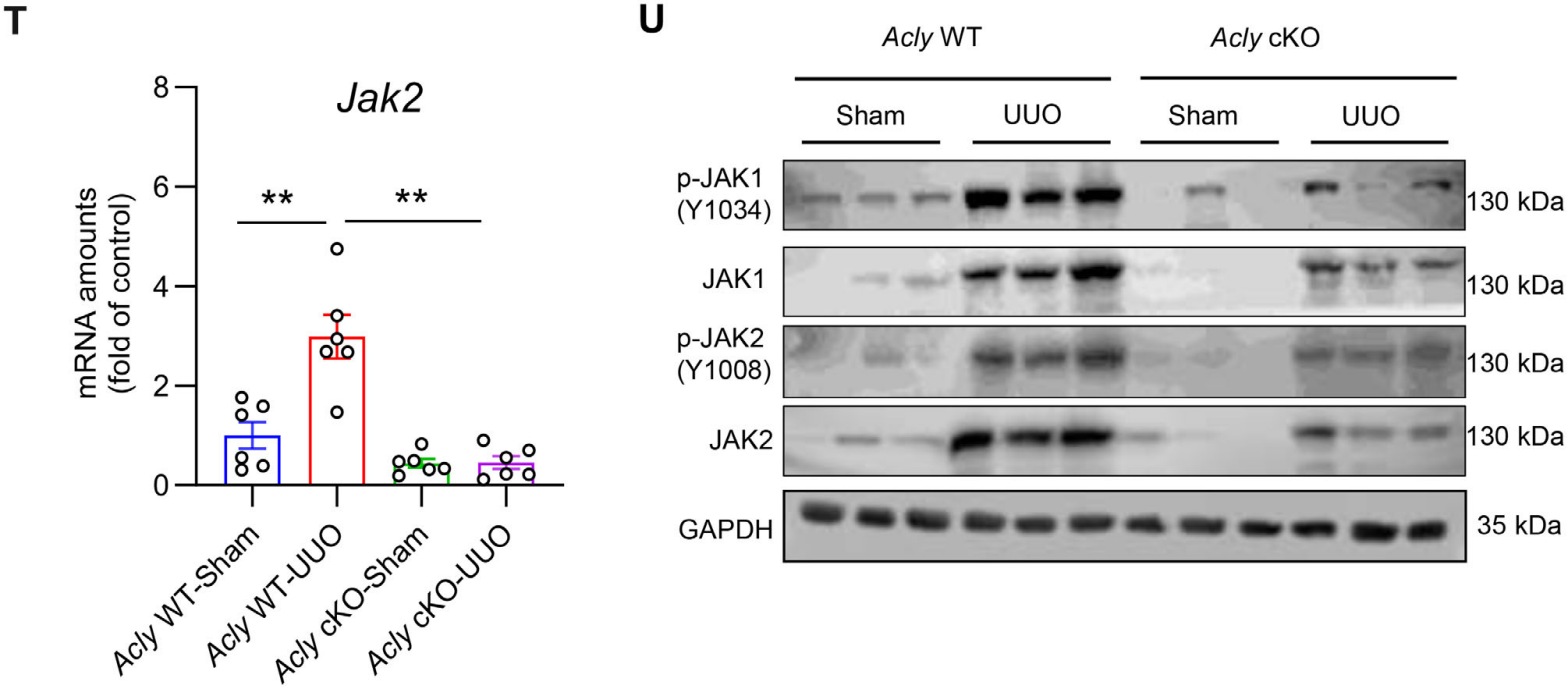

However, differential accessibility analysis revealed changes in specific regions. Approximately 13% of total peaks were classified as differentially accessible regions (DARs) between WT and KO samples, with both gains and losses in accessibility (Figure 4E,F). Regions that showed decreased accessibility in *Acly*‐deficient kidneys were enriched for gene ontology (GO) terms related to cytoskeletal organization and cell‐substrate adhesion (Figure 4G). Kyoto Encyclopedia of Genes and Genomes (KEGG) enrichment analysis further revealed associations with key fibrotic signaling pathways, including PI3K‐AKT, MAPK, focal adhesion, and citrate metabolism (Figure 4H). Together, these findings suggest that ACLY contributes to fibrosis‐associated chromatin remodeling at specific regulatory regions.

To assess the relevance of ACLY‐dependent chromatin changes, we compared genes linked to decreased accessibility in *Acly* cKO mouse kidneys with genes that exhibit increased accessibility in proximal tubule cells from CKD patients (Figure 4I). As reported in a recent single‐nucleus ATAC‐seq analysis of more than 60,000 human kidney cells, CKD is characterized by disease‐associated accessibility gains in metabolic genes predominantly expressed in proximal tubules [23]. A total of 172 genes (15.2%) that lost accessibility in *Acly* cKO mice overlapped with genes exhibiting disease‐associated chromatin opening in human CKD (Figure 4J), highlighting a conserved fibrosis‐associated epigenetic signature involving ACLY‐regulated chromatin regions.

To investigate the functional relevance of chromatin regions regulated by ACLY, we focused on genomic regions that exhibited reduced chromatin accessibility in *Acly* cKO kidneys. Since ACLY promotes acetyl‐CoA production and histone acetylation, particularly H3K27ac, its activity is expected to support chromatin opening and transcriptional activation at key regulatory loci. We therefore hypothesized that regions with decreased accessibility in *Acly* cKO kidneys represent ACLY‐dependent chromatin opening events, potentially linked to protection against fibrosis. Consistent with this hypothesis, analysis of regions with reduced chromatin accessibility in *Acly* cKO kidneys revealed significant enrichment at loci associated with renal fibrosis. Specifically, decreased accessibility was observed at multiple collagen genes (including *Col5a1*, *Col4a5*, and *Col27a1*), hyaluronan synthase family members (such as *Has3*), and the extracellular matrix gene *Fn1* (Figure S7). These findings indicate that ACLY deficiency restricts chromatin opening at key profibrotic gene loci, thereby limiting activation of fibrotic transcriptional programs and potentially contributing to protection against renal fibrosis. Then we mapped the genes associated with these regions to the annotated gene sets in the nCounter Human Fibrosis V2 Panel (NanoString Technologies, Seattle, WA), which includes 760 fibrosis‐related genes categorized into four biological themes: inflammation, proliferation, initiation, and modification (Figure 4K). We found that genes linked to ACLY‐dependent open chromatin regions were enriched in the inflammation module, followed by proliferation and initiation pathways (Figure 4L). This suggests that ACLY maintains chromatin accessibility at regulatory regions of inflammation‐ and proliferation‐related genes, possibly contributing to transcriptional programs that promote fibrotic progression. We then cross‐referenced the 172 shared DAR‐associated genes (identified from both *Acly* cKO mouse kidneys and CKD patient proximal tubules) with the 760‐gene NanoString Human Fibrosis V2 Panel. This analysis highlighted several candidate regulators, including *Jak1* and *Jak2*, which are key members of fibrosis‐associated gene modules. Notably, both *Jak1* and *Jak2* were annotated across multiple functional categories‐inflammation, proliferation, and modification‐emphasizing their relevance to injury‐activated fibrotic programs (Figure 4M). To define the chromatin basis of their regulation, we examined H3K27ac‐marked regulatory regions at the *Jak1* and *Jak2* loci using published ChIP‐seq datasets [24]. Genome browser visualization revealed prominent H3K27ac peaks at enhancer and promoter regions of *Jak1* and *Jak2*, which were notably reduced in *Acly*‐deficient kidneys (Figure 4N,O). To further validate the epigenetic regulation at these loci, we performed chromatin immunoprecipitation followed by quantitative PCR (ChIP‐qPCR) targeting the promoter regions of *Jak1*, *Jak2*, and the control locus *Rpl30*. ChIP‐qPCR analysis revealed reduced H3K27ac enrichment at the *Jak1* and *Jak2* promoters in *Acly*‐deficient tubular epithelial cells compared with WT cells under TGF‐β stimulation, whereas H3K27ac levels at the *Rpl30* promoter remained unchanged, supporting locus‐specific regulation of histone acetylation by *Acly (*Figure 4P–R).To functionally validate the conserved role of JAK1 and JAK2 in ACLY‐mediated chromatin regulation and fibrosis, we examined their expression in the UUO model. Consistent with the cross‐species chromatin analysis, *Jak1* and *Jak2* mRNA levels were increased in fibrotic WT kidneys but markedly suppressed in *Acly*‐deficient mice (Figure 4S,T). Western blot further confirmed increased phosphorylation of JAK1 and JAK2 in WT UUO kidneys, which was largely attenuated in ACLY‐deficient kidneys (Figure 4U; Figure S8A–D). Because STAT1 is a key downstream transcription factor of the JAK‐STAT signaling pathway, we next examined STAT1 activation in UUO kidneys. Both total and phosphorylated STAT1 were markedly increased in WT UUO mice but significantly attenuated in *Acly* cKO mice (Figure S8E,F). Consistently, IL‐6 expression was markedly elevated in WT UUO kidneys but significantly reduced in *Acly* cKO mice, consistent with the attenuation of JAK–STAT–mediated inflammatory signaling (Figure S8G,H). Together, these findings support the notion that ACLY contributes to renal fibrosis, at least in part, by regulating the JAK‐STAT signaling pathway.

### 2.5 Pharmacological ACLY Inhibition Mitigates Renal Fibrosis in UUO Mouse Model viaHistone Acetylation and JAK1/2 Signaling

**FIGURE 5:**
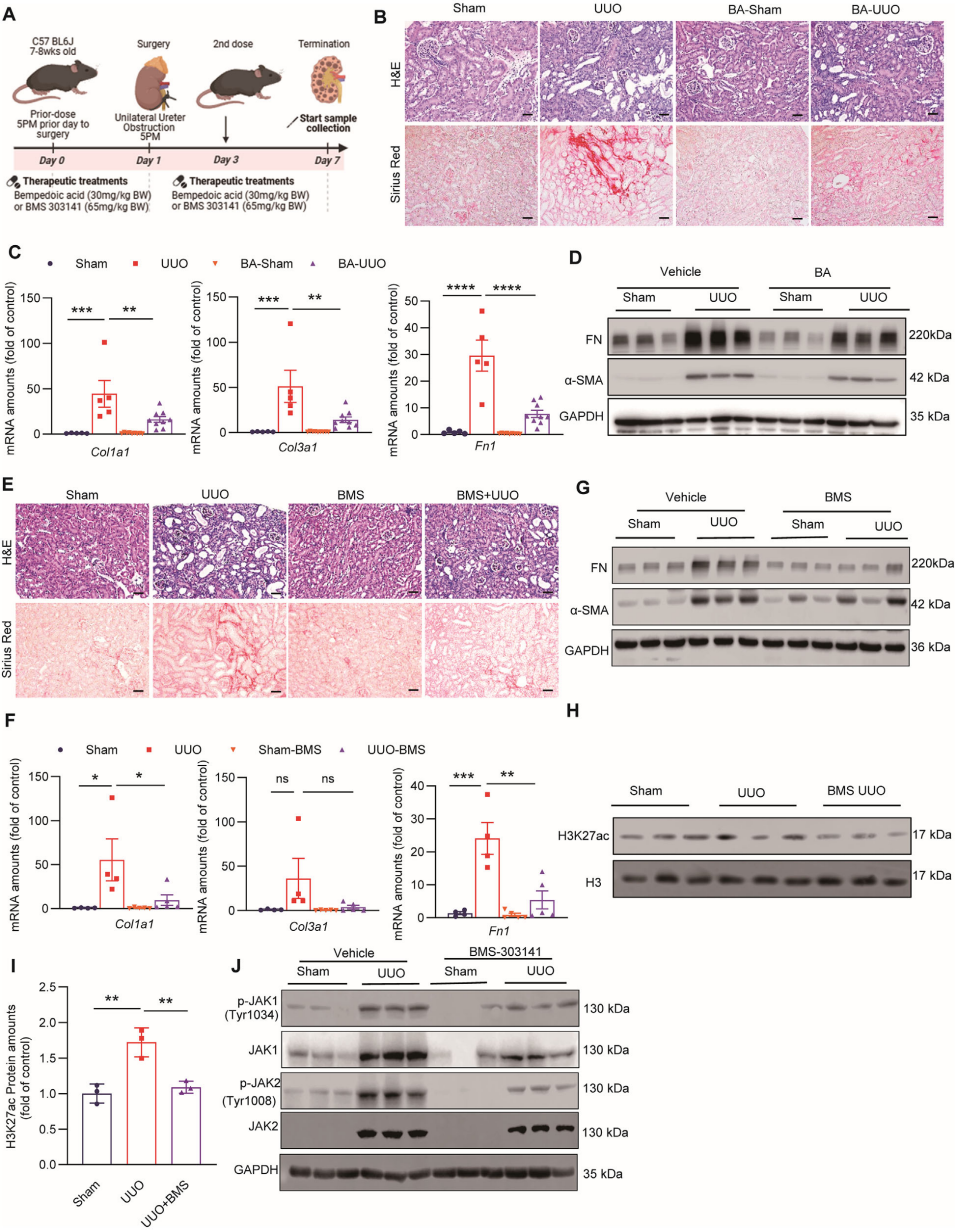
Pharmacological inhibition of *Acly* attenuates renal fibrosis in UUO mouse models. (A) Schematics of the experimental timeline. Male C57BL/6J mice (6–8 weeks) were treated with ACLY inhibitor Bempedoic acid (BA) (30 mg/kg) or BMS‐303141 (65 mg/kg) prior to and after unilateral ureteral obstruction (UUO) surgery. Kidneys were harvested on day 7 after UUO for analysis. (B) Representative H&E (top) and Sirius Red (bottom) staining of kidney sections. UUO induced severe tubular injury and interstitial collagen deposition, both of which were alleviated by BA treatment. Scale bars, 50 µm. (C) Relative mRNA levels of fibrotic genes (*Col1a1, Col3a1, Fn1*) in UUO kidneys treated with or without BA, fibrotic gene expression was lower in UUO mice treated with BA compared with UUO mice treated with vehicle. ^**^ *p* < 0.01, ^***^ *p* < 0.001, ^****^ *p* < 0.0001, n = 5–9. (D) Western blot analysis of fibronectin (FN) and α‐SMA protein levels in the UUO model treated with or without BA. (E) Representative H&E (top) and Sirius Red (bottom) staining of kidney sections from mice subjected to sham surgery or UUO, with or without BMS‐303141 treatment. Scale bars, 50 µm. (F) Relative mRNA levels of fibrotic genes (*Col1a1, Col3a1, Fn1*) in UUO kidneys treated with or without BMS‐303141, fibrotic gene expression was lower in UUO mice treated with BMS‐303141 compared with UUO mice treated with vehicle. ^*^ *p* < 0.05, ^**^ *p* < 0.01, ^***^ *p* < 0.001, n = 4–5. (G) Representative western blots of FN and α‐SMA in kidney lysates from mice subjected to sham surgery or UUO, with or without BMS‐303141 treatment. (H) Representative immunoblots of histone H3 lysine 27 acetylation (H3K27ac) in kidneys from vehicle‐ or BMS‐303141 treated mice following UUO. (I) Quantification of H3K27ac levels from (H). ^**^ *p* < 0.01, n = 3. (J) Representative western blots of total and phosphorylated JAK1 and JAK2 in kidney tissues from sham‐operated or UUO mice, with or without BMS‐303141 treatment. Data represent mean ± SEM. One‐way ANOVA with Tukey's multiple comparisons test for C, F, and I.

Building on the protective effects observed with genetic deletion of *Acly*, we next tested whether pharmacological inhibition of ACLY could similarly attenuate kidney fibrosis. Two ACLY inhibitors were used: bempedoic acid (BA), an FDA‐approved drug for hyperlipidemia, and BMS‐303141, a selective small‐molecule inhibitor commonly used in preclinical studies. Mice subjected to UUO‐induced renal fibrosis were treated with either BA or BMS‐303141, administered once prior to surgery and again on day 3 post‐injury (Figure 5A).

Histological analysis showed that BA treatment reduced tubular injury and collagen deposition in UUO kidneys, as assessed by H&E and Sirius Red staining (Figure 5B; Figure S9A). Quantitative PCR revealed decreased expression of *Col1a1* and *Fn1*, with a more modest effect on *Col3a1* (Figure 5C). Western blot confirmed lower levels of fibronectin and α‐SMA protein in kidneys from BA–treated mice (Figure 5D; Figure S9B,C). These anti‐fibrotic effects were consistently replicated in mice treated with BMS‐303141 (Figure 5E–G; Figure S9D–F), supporting the therapeutic relevance of ACLY inhibition in renal fibrosis.

Importantly, treatment with BMS‐303141 reduced H3K27ac levels in UUO kidneys (Figure 5H,I), indicating that ACLY activity is required to maintain elevated histone acetylation during fibrosis progression. In parallel, we observed reduced expression and phosphorylation of JAK1 and JAK2 following BMS‐303141 treatment (Figure 5J; Figure S10), suggesting that ACLY inhibition may attenuate fibrosis by modulating JAK‐STAT signaling.

Together, these findings position ACLY as an important metabolic–epigenetic regulator of fibrotic signaling in the kidney and support the potential for ACLY inhibition as a therapeutic strategy in CKD.

### 2.6 ACLY Activation and Increased H3K27ac and JAK1/2 Expression in Human CKD Kidneys

**FIGURE 6:**
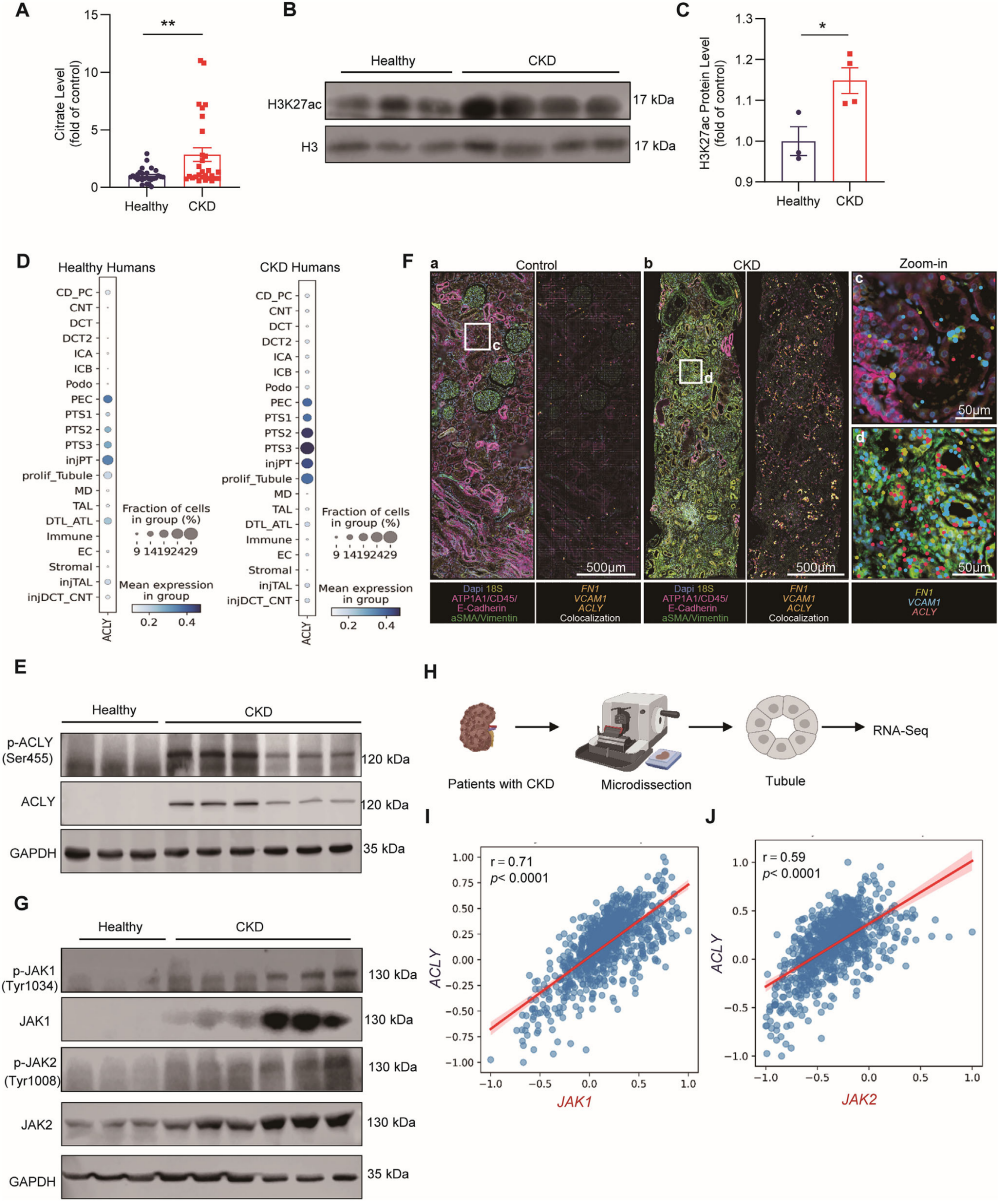
ACLY activation and increased H3K27ac and JAK1/2 expression in human CKD kidneys. (A) Relative quantification of citrate levels in human chronic kidney disease (CKD), including hypertensive nephropathy and diabetic kidney disease, compared with healthy controls. ^**^ *p* < 0.01, n = 28. (B) Western blots of histone H3 lysine 27 acetylation (H3K27ac) and total histone H3 in healthy and CKD human kidney cortical samples. (C) Quantification of H3K27ac normalized to H3 from (B). ^*^ *p* < 0.05, n = 3–4. (D) Single‐nucleus RNA‐seq analysis of human kidney biopsies showing *Acly* expression in proximal tubular segments in healthy and CKD patients, including hypertensive nephropathy and diabetic kidney disease. CD_PC: Collecting Duct Principal Cells, CNT: Connecting Tubule Cells, DCT: Distal Convoluted Tubule Cells, DCT2: Distal Convoluted Tubule Segment 2, ICA: Intercalated Cells, Type A, ICB: Intercalated Cells, Type B, Podo: Podocytes, PEC: Parietal Epithelial Cells, PTS1: Proximal Tubule Segment 1, PTS2: Proximal Tubule Segment 2, PTS3: Proximal Tubule Segment 3, injPT: Injured Proximal Tubule Cells, prolif_Tubule: Proliferating Tubular Cells, MD: Macula Densa, TAL: Thick Ascending Limb, DTL_ATL: Descending Thin Limb/Ascending Thin Limb, Immune: Immune Cell Populations, EC: Endothelial Cells, Stromal: Stromal/Fibroblast Cells, injTAL: Injured Thick Ascending Limb, injDCT_CNT: Injured Distal Convoluted Tubule/Connecting Tubule Cells. (E) Representative Western blot analysis of total ACLY and phosphorylated ACLY (Ser455) in kidney samples from healthy controls and patients with CKD due to hypertensive nephropathy. Healthy subjects (eGFR > 90 mL/min/1.73 m^2^), whereas the CKD group showed reduced kidney function with a mean eGFR of approximately 50 mL/min/1.73 m^2^. (F) Representative spatial transcriptomic images of human kidney sections from healthy donors (a, Control) and patients with CKD (b, CKD). The left images in each group show spatial expression of epithelial, immune, and stromal cell markers, including *ATP1A1* (proximal tubules), *CD45* (immune cells), *E‐Cadherin* (epithelial cells), *α‐SMA/Vimentin* (myofibroblasts), overlaid with DAPI and 18S rRNA signals. The right images highlight co‐localization of *ACLY* with fibrotic markers *FN1* and *VCAM1*. Scale bars, 500 µm. Zoom‐in views revealed increased ACLY expression in tubular epithelial cells from CKD kidneys compared with controls. Scale bars (a,b), 500 µm, Scale bars (Zoom‐in), 50 µm. (G) Western blot analysis of phosphorylated and total JAK1 and JAK2 in kidney samples from healthy controls and patients with hypertensive nephropathy–associated CKD. Healthy controls exhibited preserved renal function (eGFR > 90 mL/min/1.73 m^2^), whereas CKD patients showed impaired kidney function with a mean eGFR of approximately 50 mL/min/1.73 m^2^. (H) Schematic illustrating workflow for RNA‐seq analysis from microdissected renal tubules of CKD patients. (I,J) Correlation between kidney ACLY expression (y‐axis) and JAK1 (I) or JAK2 (J) expression (x‐axis) in 843 microdissected tubule transcriptomes from healthy individuals and patients with CKD. (Pearson correlation, *p* < 0.0001 for both). Data represent mean ± SEM. Unpaired two‐tailed *t*‐test for A, C.

To determine whether ACLY activation and histone hyperacetylation also occur in human CKD, we analyzed kidney tissue samples from patients with CKD and healthy controls. Citrate levels were elevated in CKD samples, consistent with findings in mouse models (Figure 6A). Western blot revealed higher H3K27ac levels in CKD patient kidneys compared to healthy controls (Figure 6B,C), indicating enhanced histone acetylation in human disease.

Single‐nucleus RNA‐seq analysis of human kidney datasets showed that ACLY expression is enriched in proximal tubule segments under baseline conditions and is higher in injured and proliferating proximal tubular cells in CKD patients (Figure 6D), mirroring mouse transcriptomic profiles. Western blot further confirmed higher ACLY phosphorylation at Ser455 in CKD kidneys, suggesting a link between ACLY activation and disease severity (Figure 6E; Figure S11A,B). Spatial transcriptomic analysis revealed that ACLY was higher in proximal tubule regions of CKD kidneys, where it co‐localized with pro‐inflammatory markers VCAM1 and FN1, high‐resolution zoom‐in images demonstrated marked upregulation of ACLY within renal tubular epithelial cells in CKD kidneys, consistent with enhanced metabolic‐epigenetic activation in injured tubules (Figure 6F). In kidney samples from patients with CKD, ACLY and JAK1/2 protein levels were higher than in healthy controls and increased with worsening renal function (Figure 6G; Figure S11C–F). Transcriptomic analysis of microdissected tubules further demonstrated a strong positive correlation between *ACLY* and *JAK1* (r = 0.71) and *JAK2* (r = 0.59) expression (Figure 6H–J), The strong association between ACLY and JAK1/2 expression in human CKD tubules further supports a conserved regulatory link between ACLY and JAK–STAT signaling during human kidney fibrosis.

## 3 Discussion

In this study, we investigate how metabolic dysregulation can produce lasting changes in chromatin state and gene expression that contribute to chronic kidney disease (CKD progression. Focusing on CKD, we define a working model that links cellular metabolism, epigenetic remodeling, and inflammatory signaling in renal tubular cells. By integrating epigenomic profiling, metabolomics, genetic models, pharmacologic inhibition, and human kidney tissue analysis, we identify ACLY as a metabolic–epigenetic integrator in proximal tubules. Our data support a pathway in which ACLY converts citrate into acetyl‐CoA, thereby sustaining histone H3K27 acetylation at pro‐inflammatory gene loci, including *Jak1* and *Jak2*, and contributing to JAK‐STAT‐associated inflammatory and fibrotic transcriptional programs. Tubule‐specific deletion or pharmacologic inhibition of ACLY suppresses this axis, leading to reduced chromatin accessibility at these loci, lower cytokine production, and attenuated renal fibrosis. These findings provide a mechanistic framework that links nutrient sensing to epigenetic remodeling and chronic inflammation in CKD.

While epigenetic changes have long been proposed in CKD [25], no unbiased profiling has been performed due to technical difficulties in histone modification analysis. To uncover conserved epigenetic changes in kidney fibrosis, we performed quantitative histone tail proteomics in two widely used murine models—FA and UUO [26]. Across both models, we identified a consistent and selective increase in H3K27ac, a modification associated with transcriptionally active enhancers and promoters. This reproducible enrichment suggests that H3K27ac represents a prominent epigenetic feature of renal fibrogenesis, independent of injury type. We next sought to identify the metabolic drivers of this acetylation pattern. Given that acetate can also serve as an upstream carbon source for acetyl‐CoA via ACSS2, we quantified acetate levels in both fibrotic kidney tissues and in circulation. ACSS2 protein expression was reduced in UUO and was not further altered in *Acly*‐deficient UUO kidneys, arguing against a major compensatory role for the acetate–ACSS2 pathway in sustaining acetyl‐CoA production during fibrosis. In contrast, both fibrosis models exhibited citrate accumulation, a hallmark of disrupted mitochondrial metabolism. Both fibrosis models exhibited citrate accumulation, a hallmark of disrupted mitochondrial metabolism. Because citrate is the precursor for cytosolic acetyl‐CoA production via ACLY [22, 27, 28], we hypothesized that increased ACLY activity fuels H3K27ac at proinflammatory and profibrotic gene loci. Supporting this, we observed elevated levels of acetyl‐CoA and upregulation of ACLY in fibrotic kidneys, along with strong spatial enrichment in proximal tubule epithelial cells—a metabolically active and injury‐prone segment. These data establish a mechanistic link between metabolic stress, citrate flux, and ACLY‐mediated chromatin remodeling, and support a role for ACLY as a metabolic–epigenetic gatekeeper in CKD. While our data highlight histone H3K27 acetylation as the dominant epigenetic output of ACLY activation in fibrotic kidneys, ACLY‐derived acetyl‐CoA may also support acetylation of specific non‐histone proteins involved in fibrotic and inflammatory signaling pathways. The extent to which these select non‐histone acetylation events contribute to CKD pathogenesis remains largely unexplored and warrants further investigation.

ACLY has recently been implicated in fibrotic remodeling in other organs, including cardiac and vascular tissues, where ACLY‐derived acetyl‐CoA promotes H3K27 acetylation at profibrotic gene loci and pharmacologic ACLY inhibition limits fibrogenesis. Our study extends these findings to the kidney and adds several elements that are specific to renal disease. First, unbiased histone tail proteomics in two complementary kidney fibrosis models identify H3K27ac as a predominant histone modification associated with renal fibrogenesis. Second, we link citrate accumulation, ACLY activation, and H3K27ac to changes in chromatin accessibility and expression of JAK–STAT pathway components in proximal tubules. Third, we validate this axis in human CKD using metabolomics, spatial transcriptomics, and microdissected tubular transcriptomes, where ACLY activation and JAK1/2 expression associate with loss of kidney function. Together, these findings position ACLY‐dependent epigenetic remodeling as a conserved but organ‐specific amplifier of fibrotic signaling and highlight its relevance in human CKD.

Our data suggest that ACLY modulates chromatin accessibility at pro‐inflammatory loci. Integrative analysis of ATAC‐seq and H3K27ac ChIP‐seq datasets revealed reduced chromatin accessibility at H3K27ac‐marked regulatory regions of *Jak1* and *Jak2* in *Acly*‐deficient kidneys, accompanied by decreased JAK1/2 expression and reduced JAK1/2 phosphorylation in fibrotic kidneys. Consistently, STAT1 activation and IL‐6 expression were also diminished following *Acly* deletion. Together, these findings suggest that ACLY‐dependent enhancer activation promotes JAK–STAT–mediated inflammatory signaling. However, we did not directly manipulate JAK–STAT signaling downstream of ACLY, and additional cell‐type–specific and rescue experiments will be required to establish the extent to which the JAK‐STAT axis is necessary and sufficient for ACLY‐mediated fibrotic progression. Within these limits, our data are consistent with a model in which ACLY does not act as a broad global chromatin modifier, but rather preferentially supports activation of disease‐relevant inflammatory gene networks in this setting. The chromatin changes observed in *Acly*‐deficient kidneys were accompanied by reduced IL‐66 expression, implicating ACLY in the activation of the JAK–STAT signaling cascade—a critical driver of chronic inflammation and fibrosis. We also found that ACLY is phosphorylated at the activating Ser455 site in CKD, likely via AKT [29], and localized predominantly to proximal tubules, which are both metabolically active and highly injury‐prone. Taken together, these findings extend ACLY's role beyond a metabolic enzyme and support its function as a metabolic–epigenetic regulator that links mitochondrial dysfunction and metabolic stress to chromatin remodeling and downstream inflammatory signaling. Through its regulation of JAK1/2 and IL‐6, ACLY connects nutrient sensing to cytokine‐driven fibrosis, establishing a metabolism‐to‐epigenome‐to‐inflammation axis that likely contributes to sustained disease progression in CKD. This expanded role raises the possibility that ACLY‐dependent chromatin remodeling may contribute to aspects of epigenetic “memory,” and that this process is dynamically regulated and potentially reversible, opening new avenues for therapeutic intervention in chronic fibrotic diseases.

The clinical relevance of our findings is underscored by the availability of ACLY inhibitors already in therapeutic use. We demonstrate that pharmacological inhibition of ACLY, using either the selective small‐molecule BMS‐303141 or the FDA‐approved drug BA, phenocopies the protective effects of genetic *Acly* deletion, suppressing H3K27 acetylation, reducing JAK1/2 expression, and mitigating fibrosis in two distinct mouse models. Importantly, BA is currently approved for the treatment of hyperlipidemia, with a favorable safety profile and oral bioavailability, raising the exciting possibility of repurposing this agent for CKD therapy. In support of this translational direction, human CKD kidney biopsies showed higher ACLY expression and phosphorylation, elevated H3K27ac levels, and upregulation of JAK1/2, confirming that this pathway is activated in human disease. Although side effects such as hyperuricemia and tendon injury may warrant caution in CKD populations, our results provide a compelling mechanistic rationale for targeting ACLY in kidney fibrosis and support the clinical evaluation of ACLY inhibitors, particularly BA, as a tractable strategy to slow or halt CKD progression.

This study has several limitations. First, we focused on acute and subacute injury models (FA and UUO), which produce robust fibrosis but do not capture the full spectrum of common human CKD etiologies such as long‐standing diabetes and hypertension. Whether the citrate–ACLY–H3K27ac–JAK axis operates in the same way in more indolent metabolic models remains to be tested. Second, while these data support a dominant role for ACLY in the tubular compartment, we cannot exclude potential context‐dependent contributions of ACLY within glomerular and other cells. Importantly, our genetic models selectively target tubular epithelium, and epigenetic regulation by ACLY in glomerular populations was not directly examined in this study. Future investigations employing glomerulus‐specific genetic manipulation and higher‐resolution, cell type–resolved epigenomic profiling will be required to determine whether ACLY also modulates chromatin states and functional responses within the non‐tubular cell types, such as the glomerular compartment, fibroblasts, endothelial cells, or infiltrating immune cells. Spatial and single‐cell analyses support a prominent role for proximal tubules, but future studies will be needed to dissect cell‐type–specific ACLY functions in vivo. Third, although ACLY expression and metabolic reprogramming were most pronounced in proximal tubules, our genetic model (*Ksp*‐Cre; *Acly* ^fl/fl^) drives recombination broadly across the renal tubular epithelium, including distal nephron and collecting duct segments. Thus, we cannot fully exclude contributions from non‐proximal tubular segments to the observed chromatin and transcriptional changes. Nevertheless, spatial transcriptomics, metabolic profiling, and chromatin analyses consistently identify proximal tubules as the dominant site of ACLY activation and H3K27ac remodeling, supporting a primary role for this segment in mediating the observed phenotype. Fourth, our evidence for JAK–STAT involvement is associative and based on chromatin accessibility, gene expression, and phosphorylation status; we did not perform genetic or pharmacologic rescue experiments in the JAK–STAT pathway. Finally, while ACLY inhibition with bempedoic acid and BMS‐303141 phenocopied genetic *Acly* deletion, these agents may have off‐target or ACLY‐independent effects, and their efficacy and safety in CKD will require dedicated clinical studies. Fifth, we attempted to profile histone modification landscapes in human kidney samples using a proteomics‐based approach. However, due to substantial inter‐patient heterogeneity, along with variability in tissue availability, ischemic time, and sample preservation, we were unable to obtain reproducible and robust histone modification profiles across human specimens. These technical and biological limitations precluded definitive interpretation of the proteomics data. We therefore focused our human analyses on reproducible immunohistochemical and biochemical assessments.

This study also highlights several important areas for future investigation. First, the paracrine effects of ACLY‐driven tubular IL‐6 production—including its influence on immune cell recruitment, activation, and fibrogenesis—remain to be defined using co‐culture systems or in vivo lineage‐tracing approaches. Second, although we validated the ACLY–H3K27ac–JAK axis using bulk and spatial transcriptomics in human CKD kidneys, future single‐cell epigenomic studies will be needed to define cell‐type–specific contributions and to uncover potential heterogeneity across disease stages and nephron segments. The role of ACLY in non‐tubular cell populations, including infiltrating immune cells and resident fibroblasts, also warrants further investigation. Despite these uncertainties, our findings provide robust evidence that ACLY functions as a key metabolic–epigenetic regulator of kidney fibrosis.

In conclusion, we identify ACLY as an important driver of chromatin remodeling and inflammatory gene activation in CKD. By linking citrate metabolism to histone H3K27 acetylation and JAK‐STAT signaling, our findings reveal a mechanistic framework by which metabolic dysregulation can fuel epigenetic reprogramming and fibrotic progression. This work suggests that metabolic–epigenetic reprogramming is not merely a downstream consequence of injury, but also a potentially modifiable contributor to CKD pathogenesis. The discovery of a tractable citrate–ACLY–H3K27ac–JAK axis offers new avenues for therapeutic intervention and supports the potential repurposing of ACLY inhibitors, such as BA, for the treatment of fibrotic kidney disease.

## 4 Experimental Section

### 4.1 Animal Studies

Tubule‐specific *Acly* deletion was generated by crossing *Acly* ^fl/fl^ mice with *Ksp*‐Cre (*Cdh*16‐Cre) mice (Jackson Lab, stock#012237). *Ksp*‐Cre mediates recombination broadly in renal tubular epithelial cells—including proximal tubules, distal tubules, and collecting ducts. Littermate *Acly* ^fl/fl^ mice lacking Cre were used as controls. Primer sequences used for genotyping are listed in Table S1.

Construction of CKD mouse models was performed as previously described in the literature [17]. Mice (6–8 weeks old) were randomly assigned to experimental groups and maintained with ad libitum access to food and water. To interrogate distinct nephron compartments, we employed two complementary injury models: folic acid (FA) nephropathy, and unilateral ureteral obstruction (UUO),. FA nephropathy was induced by a single intraperitoneal injection of FA dissolved in sodium bicarbonate (250 mg/kg). UUO was performed by ligating the right ureter under anesthesia; the contralateral kidney served as the sham control. Mice were euthanized 7 days after FA administration or surgery.

For therapeutic studies, BMS‐303141 was administered intraperitoneally at 65 mg/kg body weight 1 day before UUO surgery and a second time on day 3 after surgery, and mice were euthanized on day 7 for tissue and serum collection. For bempedoic acid treatment, mice received 30 mg/kg body weight by oral gavage once daily for 7 consecutive days, starting 1 day before UUO surgery (day 0) and continuing through day 6; kidneys were harvested on day 7. Control animals received the corresponding vehicle on the same schedule. Doses and schedules for BMS‐303141 and bempedoic acid were selected based on prior in vivo ACLY inhibition studies and adjusted in pilot experiments to achieve robust pathway modulation without overt systemic toxicity.

Mice were assigned to experimental groups based on genotype or randomly allocated to treatment groups. Blinding was not possible due to the nature of the UUO surgery and genotype‐based grouping. No experimental units or data points were excluded.

### 4.2 Histone Extraction and LC‐MS/MS Based Quantification

Kidneys were harvested from healthy control mice and from CKD models, including FA–induced nephropathy and UUO, followed by histone extraction using acid precipitation. Briefly, freshly isolated kidney cortices were homogenized, and nuclei were isolated. Histones were extracted by incubating nuclear pellets in 0.4 NH_2_SO_4_, followed by precipitation with trichloroacetic acid and washing with acetone. The extracted histones were subjected to liquid chromatography–tandem mass spectrometry (LC‐MS/MS) using the EpiProfiler 2.0 platform to profile post‐translational modifications. Differential histone acetylation levels, including H3K27ac, were quantified and analyzed using log_2_ fold‐change and –log_10_ (*P* value) based volcano plots.

### 4.3 RNA Scope In Situ Hybridization

Kidney tissues were fixed in 4% paraformaldehyde, embedded in paraffin, and sectioned at 5 µm thickness. RNAscope multiplex fluorescence in situ hybridization (FISH) was performed using probes targeting *Acly* and *Lrp2* transcripts (Advanced Cell Diagnostics) following the manufacturer‘s instructions. Hybridization signals were visualized using confocal microscopy. Co‐localization of *Acly* (magenta) with *Lrp2* (cyan), a proximal tubular marker, was assessed in healthy mouse kidneys.

### 4.4 Western Blotting

For Western blot analysis, kidney lysis was conducted using blue buffer (Cell Signaling Technology, #56036S). Following centrifugation at 12 000 g for 15 min Subsequently, cell lysates were combined with SDS‐PAGE loading buffer, resolved by 10% SDS‐PAGE, and transferred onto PVDF membranes (Millipore). The membranes were subsequently subjected to overnight incubation with primary antibodies, including H3K27ac (Abcam, #ab4729), total histone H3 (Abcam, #ab176842), ACLY (Abcam, ab40793), p‐ACLY (Invitrogen, PA5‐105112), anti‐α‐SMA (Sigma, #A5228), anti‐fibronectin (Abcam, #ab2413), JAK1(Cell Signaling Technology, CAT#3344T), p‐JAK1 (Cell Signaling Technology, CAT 74129T), JAK2 (Cell Signaling Technology, CAT#3230T), p‐JAK2 (Cell Signaling Technology, CAT#8082T), STAT1(Cell Signaling Technology, CAT#14994), p‐STAT1(Cell Signaling Technology, CAT#9167) and GAPDH (Cell Signaling Technology, CAT#2118S), IL‐6 (Proteintech, CAT#21865‐1‐AP) all used at a dilution of 1:1000. This was followed by a 1‐h incubation with secondary antibodies at room temperature. Finally, the membranes were treated with SuperLumia ECL Plus HRP Substrate Reagent, and signals emanating from immunoreactive bands were detected utilizing a Chemiluminescent Imaging System.

### 4.5 Quantitative Real‐Time PCR

Total RNA was extracted from mouse kidneys using TRIzol reagent (Invitrogen) and reverse‐transcribed using a high‐capacity cDNA reverse transcription kit (Thermo Fisher Scientific). Quantitative PCR was performed using SYBR Green Master Mix (Bio‐Rad) on a QuantStudio 5 Real‐Time PCR system (Applied Biosystems). Gene expression was normalized to *Gapdh* and analyzed using the ΔΔCt method. Primer sequences are listed in Table S1.

### 4.6 Metabolite Extraction and LC‐MS/MS Based Metabolomics

Metabolite detection was performed as previously described [11]. Kidneys from mice and human were collected and weighed. Snap frozen samples were sent to Metabolon. Raw data were extracted, peak‐identified, and quality‐control processed using Metabolon's hardware and software. A data normalization step was performed to correct variation resulting from instrument inter‐day tuning differences. PCA was tested to reduce the dimension of the data. The pathway analysis using MetaboAnalyst (http://www.metaboanalyst.ca) with the default setting was performed for the metabolites changed in mice and human kidney tissues. The human study was deemed exempt by the Institutional Review Board of the University of Pennsylvania as no personal identifiers were collected.

### 4.7 Quantification of Citrate and Acetyl‐CoA

Citrate concentrations in kidney tissues were quantified using a commercial Citrate Assay Kit (Abcam, ab83396) following the manufacturer's protocol. Absorbance at 570 nm was measured using a microplate reader (BioTek Synergy), and data were normalized to tissue weight or protein content.

### 4.8 Acetyl‐CoA

Acetyl‐CoA levels were determined using an Acetyl‐Coenzyme A Assay Kit (Sigma, MAK566). Tissue extracts were deproteinized using perchloric acid or spin columns, and fluorescence was measured at Ex/Em = 535/587 nm. Concentrations were interpolated from standard curves and expressed as pmol/µL.

### 4.9 H&E and Sirius Red Staining

The tissues were fixed in formalin, dehydrated by an ethanol gradient (30%, 50%, 75%, and 95%), and then submitted to the histology Core in 100% ethanol. Once the tissue was sectioned, H&E and Sirius red staining were performed. Images were acquired in an Olympus 5000 microscope with Cell Sense software. The percentage of relative fibrosis was quantified in ImageJ.

### 4.10 Primary Cell Culture Isolation

Primary tubular epithelial cells were isolated from 4–6 weeks old WT and *Acly* cKO mice kidneys. Briefly, kidneys were minced into pieces and digested in 10 mL RPMI medium containing 100 µL of Collagenase IV (1 mg/mL) for 30 min at 37°C. Afterward, collagenase IV activity was stopped by adding 100 µL of fetal bovine serum (FBS). Cells were sequentially sieved through 100, 70, and 40 µm nylon mesh and centrifuged for 10 min at 3000 rpm. The pellet was resuspended in 1 mL of sterile RBC lysis buffer and incubated for 2 min on ice. DPBS was added and centrifuged for 10 min at 3000 rpm. The pellet was then resuspended in PTECs media (RPMI 1640 supplemented with 10% FBS, 20 ng/mL hEGF, 20 ng/mL EGF, 1X ITS (insulin‐transferrin‐selenium), and 1% penicillin‐streptomycin).

### 4.11 Sub‐Cellular Fractionation

Kidney tissues were carefully homogenized in 1mL of lysis buffer (10 mM HEPES pH 7.5, 10 mM KCl, 0.1 mM EDTA, 0.5% NP‐40, 1mM DTT, and 1X protease inhibitor cocktail). Fifty microliters aliquot (5%) was taken to use as the whole cell extract. Lysed tissues were briefly vortexed and centrifuged at 12 000 g for 10 min at 4°C. The supernatant was collected as the cytoplasmic (non‐nuclear) fraction and the pellet was washed 4 times by resuspending in 200 µl of lysis buffer and spun down at 200 g for 5 min at 4°C. The nuclear pellet was then resuspended in 250 µl of Fractionation Buffer (2M sucrose, 1mM MgCl2 and 10mM Tris‐HCl pH 7.4) and centrifuged at 16 000 g for 30 min at 4°C. Supernatant was discarded and purified nuclei were washed 2 times by resuspending in 200 µl of lysis buffer and spun down at 200 g for 5 min at 4°C. Purified nuclei were then resuspended in 100 µl of Nuclear Extraction Buffer (20 mM HEPES pH 7.5, 400 mM NaCl, 1 mM EDTA, 1mM DTT, and 1X protease inhibitor cocktail) and incubated on ice for 5 min. The sample was centrifuged at 16 000 g for 5 min at 4°C and the supernatant was collected as the soluble nucleoplasm fraction. For isolation of the insoluble chromatin fraction, the pellet was resuspended in 4X Laemmli buffer and alternatively boiled at 95°C and chilled on ice 3 times to dissolve. Protein concentration was measured by nanodrop and 20 µg of protein was used for western blotting [30].

### 4.12 Human Kidney Single Nuclear ATAC‐Seq

Adult human kidney single‐nuclear ATAC seq‐data was used from previous publications [23, 31, 32]. The data can be viewed at the website (http://www.susztaklab.com/Human_snATAC/index.php).

### 4.13 Mouse Bulk ATAC‐Seq and Data Analysis

Kidney cortical tissues from WT and KO mice were freshly dissected and processed for bulk ATAC‐seq as previously described with minor modifications [33, 34]. To assess the role of ACLY in regulating chromatin accessibility during kidney injury, we performed ATAC‐seq on kidney tissues from wild‐type (WT, n = 5) and tubule‐specific *Acly* knockout (KO, n = 3) mice. All samples were generated in our laboratory, and two WT datasets (GSM4752171 and GSM4752172) were previously deposited in the GEO database [35]. Approximately 50 000 nuclei were isolated from each sample using a hypotonic lysis buffer (10 mM Tris‐HCl pH 7.4, 10 mM NaCl, 3 mM MgCl_2_, 0.1% TWEEN 20, 0.1% NP 40, and 1% BSA, followed by centrifugation and washed the nuclei pellet with wash buffer (10 mM Tris‐HCl [pH 7.4], 10 mM NaCl, 3 mM MgCl2, 1% BSA, 0.1% Tween‐20). The isolated nuclei were subjected to transposition using Tn5 transposase (Illumina Nextera DNA Library Prep Kit) at 37°C for 30 min. After purification, libraries were PCR‐amplified, size‐selected, and sequenced on an Illumina NovaSeq 6000 platform to generate 50 bp paired‐end reads.

### 4.14 ATAC Data Preprocessing and Peak Calling

Raw sequencing reads were trimmed using fastp and aligned to the mm39 reference genome using Bowtie2 (v2.3.4.3). Duplicates were removed, and BAM files were filtered to retain uniquely mapped reads. Peaks were called using MACS3 (v3.0.0) with parameters: –nomodel –extsize 200 –shift 100‐q 0.05 –call‐summits. Blacklisted regions (ENCODE mm39 blacklist) and non‐standard chromosomes were removed using BEDTools (v2.29.1). For quality control, FRiP (Fraction of Reads in Peaks) scores were calculated per sample.

### 4.15 ChIP‐qPCR

ChIP‐qPCR was conducted using the Simple ChIP Kit (CST, # 9003) following the manufacturer's standard instructions. In brief, cells were cross‐linked in 1% formaldehyde‐containing cell culture medium at room temperature for 10 min and quenched with glycine. Chromatin was sheared to ∼200‐bp fragments by Micrococcal Nuclease. H3K27ac (Abcam, ab4729) antibody and non‐immune IgG (CST, 2729) were used for ChIP. Primers used for ChIP‐qPCR are listed in Table S1.

### 4.16 Differential Accessibility and Annotation

Differentially accessible regions (DARs) between WT and KO samples were identified using DiffBind or DESeq2 based on normalized read counts. Altered peaks were defined as those with adjusted *p* < 0.05 and |log_2_ fold change| > 0.25. Peaks were annotated using ChIPseeker, showing enrichment in promoter‐proximal and distal intergenic regions. Global accessibility patterns were compared using average signal intensity, and differentially accessible peaks were visualized via a volcano plot.

### 4.17 Motif Enrichment Analysis

To identify potential transcription factors associated with global chromatin accessibility, motif enrichment analysis was performed using HOMER on the union set of accessible peaks from both WT and *Acly* ^f/f; Ksp cre^ samples. De novo motif analysis was conducted with default parameters using a background set generated by HOMER.

### 4.18 Gene Ontology and Pathway Enrichment

Genes associated with DARs were mapped using GREAT [36] and annotated with clusterProfiler for GO [37] and KEGG [38] enrichment analysis. Fibrosis‐related biological processes and pathways were enriched.

### 4.19 Spatial Transcriptomics (Xenium)

Single‐cell spatial transcriptomic profiling was performed using the Xenium In Situ platform (10x Genomics). Tissue sections (5 µm thickness) were mounted onto Xenium slides according to the manufacturer's specifications. The Human Xenium Prime 5K Pan Tissue and Pathways Panel was used and supplemented with 100 custom probes targeting genes associated with tubular identity, fibrosis, inflammation, and metabolic pathways (full gene list provided in the Methods). Slides were processed according to the Xenium Cell Segmentation workflow, which includes morphology‐based segmentation using DAPI and membrane staining to enable automated cell boundary detection. Slides were subsequently loaded onto the Xenium Analyzer for in situ transcriptomic imaging and transcript decoding. Visualization and downstream analysis were performed using Xenium Explorer (10x Genomics). In this platform, each detected transcript is represented as a spatially resolved single‐molecule dot corresponding to one RNA molecule, enabling single‐molecule resolution. Protein staining images were overlaid with transcript localization to allow direct spatial comparison between immunofluorescence signals and gene expression patterns within the same tissue section.

### 4.20 Statistics

For comparisons between two groups, statistical significance was assessed using an unpaired two‐tailed Student's *t* test. For comparisons involving three or more groups, one‐way ANOVA followed by Tukey's post hoc test was used to determine pairwise differences. Normality and variance assumptions were verified prior to parametric testing. *p* value < 0.05 was considered statistically significant. Exact *P* values, statistical tests used, and sample sizes (*n*) are provided in the figure legends.

### 4.21 Study Approval

All animal studies were approved by the IACUC of the University of Pennsylvania under protocol number 804138. Human kidney sample collection was approved by the Institutional Review Board of the University of Pennsylvania. Samples were obtained through an external honest broker in a de‐identified manner.

### 4.22 Patient Consent for Publication

No written informed consent was required, as no identifiable patient information is included in this study.

This study does not involve any clinical trial or interventional study design.

### 4.23 Data Availability 

snRNA‐Seq data are publicly available in the Susztak Laboratory Kidney Biobank (https://susztaklab.com/Human_snATAC/index.php;https://susztaklab.com/hk_genemap/snRNA). ATAC‐Sequencing data have been deposited in GEO under accession codes GSE306009. Data values for bar graphs can be found in the Supplemental Supporting Data Values file. Full blots are presented in the supplemental material. Additional details on protocols and special reagents for this study are provided by the corresponding author upon request.

### 4.24 LC‐MS/MS

Metabolomic profiling was performed using LC–MS/MS to detect and quantify kidney metabolites. Relative quantification and metabolite identification were conducted based on established protocols.

Briefly, the separation was carried out using a Waters ACQUITY UPLC system equipped with a UPLC column (2.1 × 150 mm, 1.7 µm particles, Waters ACQUITY UPLC BEH C18). Mobile phase A consisted of water with 0.1% formic acid, and mobile phase B consisted of acetonitrile:methanol (95:5, v/v) with 0.1% formic acid. The flow rate was 350 µL/min. A linear solvent gradient was applied from 20% to 90% mobile phase B over 20 min, increased to 100% B at 20.1 min, maintained until 22.0 min, and re‐equilibrated to 80% B between 22.2 and 25 min.

Metabolite detection and relative quantification were performed using mass spectrometry data processing in TargetLynx (Waters), and metabolite identification was achieved by matching retention times and MS/MS spectra against reference libraries and databases.

## Author Contributions

K.S., C.D., and D.M. conceived and designed the study. C.D, D.M., and L.L performed the in vivo and in vitro experiments, C.D, D.M., L.L, C.L., S.P., B.D., E.H., L.K., Y.H., J.L., C.K., K.K., J.W., S. M., and K.W. contributed to methodology development and provided experimental or analytical support. C.D. and D.M performed data curation and visualization. K.S. supervised the overall project, provided mentorship, and acquired funding. C.D. drafted the original manuscript. K.S. critically reviewed, edited the manuscript, and coordinated project administration. All authors approved the final version of the manuscript.

## Funding

This work was supported by the NIH (grant nos. R01DK076077, R01DK087635, P50DK114786, R01DK105821, and R01DK132630 to K. Susztak).

## Conflicts of Interest

The authors declare no conflicts of interest.

## Supporting information




**Supporting File 1**: advs75247‐sup‐0001‐SuppMat.pdf.

**Supporting File 2**: advs75247‐sup‐0002‐TableS1.xlsx.

## Data Availability

The data that support the findings of this study are available from the corresponding author upon reasonable request.
