# Supplementary material for: ACLY‐Driven Metabolic Reprogramming Promotes Histone Acetylation and Inflammation‐Associated Fibrosis in Chronic Kidney Disease
Source: Adv Sci (Weinh). 2026 Apr 16:e75247. Online ahead of print. doi: 10.1002/advs.75247 (PMC13334609; doi:10.1002/advs.75247)
Supplement: Supplementary file 1 — Supporting File 1: advs75247‐sup‐0001‐SuppMat.pdf. [file ADVS-9999-e75247-s001.pdf]

Supporting Information

ACLY-driven metabolic reprogramming promotes histone acetylation and inflammation-associated fibrosis in chronic kidney disease

*Chunxiu Du, Dhanunjay Mukhi, Lingzhi Li, Chenyu Li, Siyu Pan, Bernhard Dumoulin, Eunji Ha, Lakshmi P Kolligundla, Yanjuan Hou, Jonathan Levinsohn, Chaelin Kang, Konstantin Adrian Klötzer, Junnan Wu, Samer Mohandes, Kathryn E Wellen, Katalin Susztak**

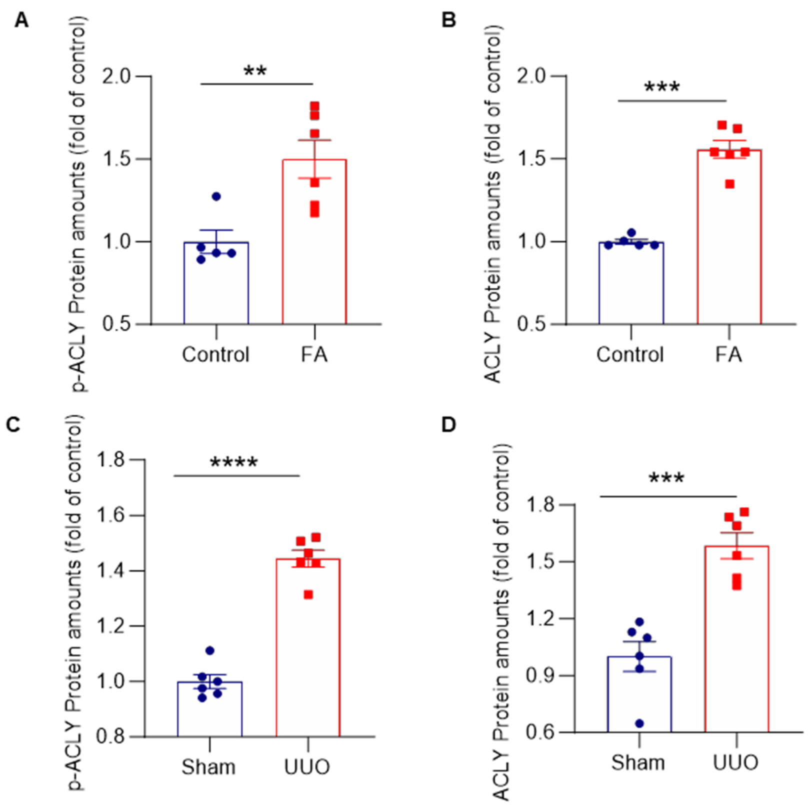

Figure S1. Quantification of ACLY and phospho-ACLY protein levels in fibrotic mouse kidneys.
(A–B) Quantification of Western blot results shown in Fig. 2I, demonstrating elevated levels of phosphorylated ACLY (p-ACLY) and total ACLY (B) in kidneys from mice subjected to folic acid (FA)–induced nephropathy compared to controls. $^{**}P < 0.01$, $^{***}P < 0.001$. n=5-6.
(C–D) Quantification of western blot results shown in Fig. 2K, showing significantly increased p-ACLY (C) and ACLY (D) protein levels in UUO kidneys compared to sham-operated mice. $^{***}P < 0.001$, $^{****}P < 0.0001$. n=5-6.
Data represent mean ± SEM. Statistical analysis was performed using unpaired two-tailed Student's *t*-test for A, B, C, D.

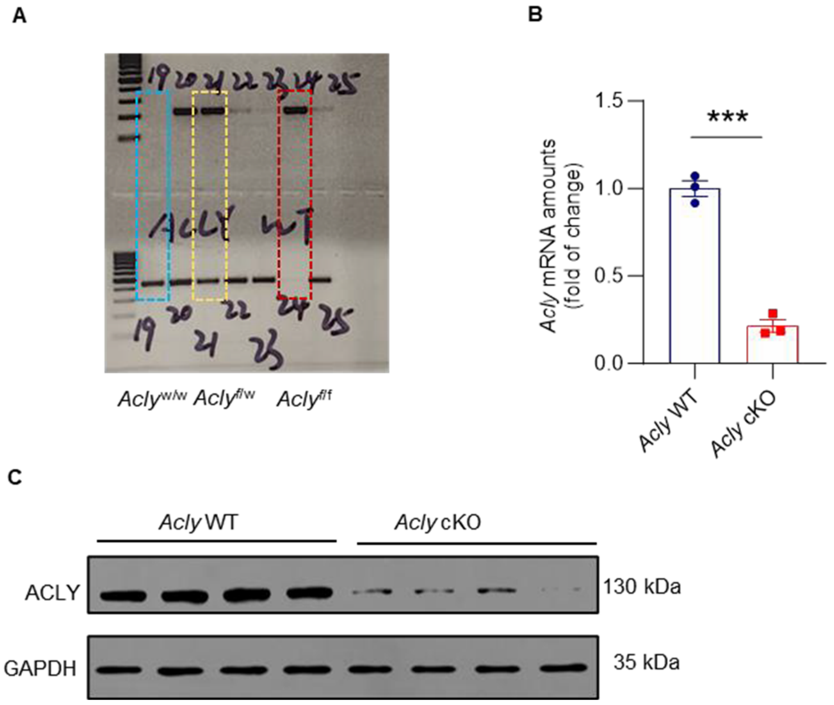

Figure S2. Generation and validation of kidney tubule–specific ACLY knockout mice.
(A) Genotyping PCR results showing wild-type (*Acly*$^{w/w}$) heterozygous (*Acly*$^{f/w}$), and homozygous floxed (*Acly*$^{f/f}$) alleles.
(B) qPCR analysis of *Acly* mRNA in primary proximal tubular cells isolated from kidneys of *Acly* WT and *Acly* cKO mice. ***$P < 0.001$. n=3.
(C) Western blots analysis of *Acly* protein in primary proximal tubular cells isolated from kidneys of *Acly* WT and *Acly* cKO mice.
Data represent mean ± SEM. Unpaired two-tailed *t*-test for B.

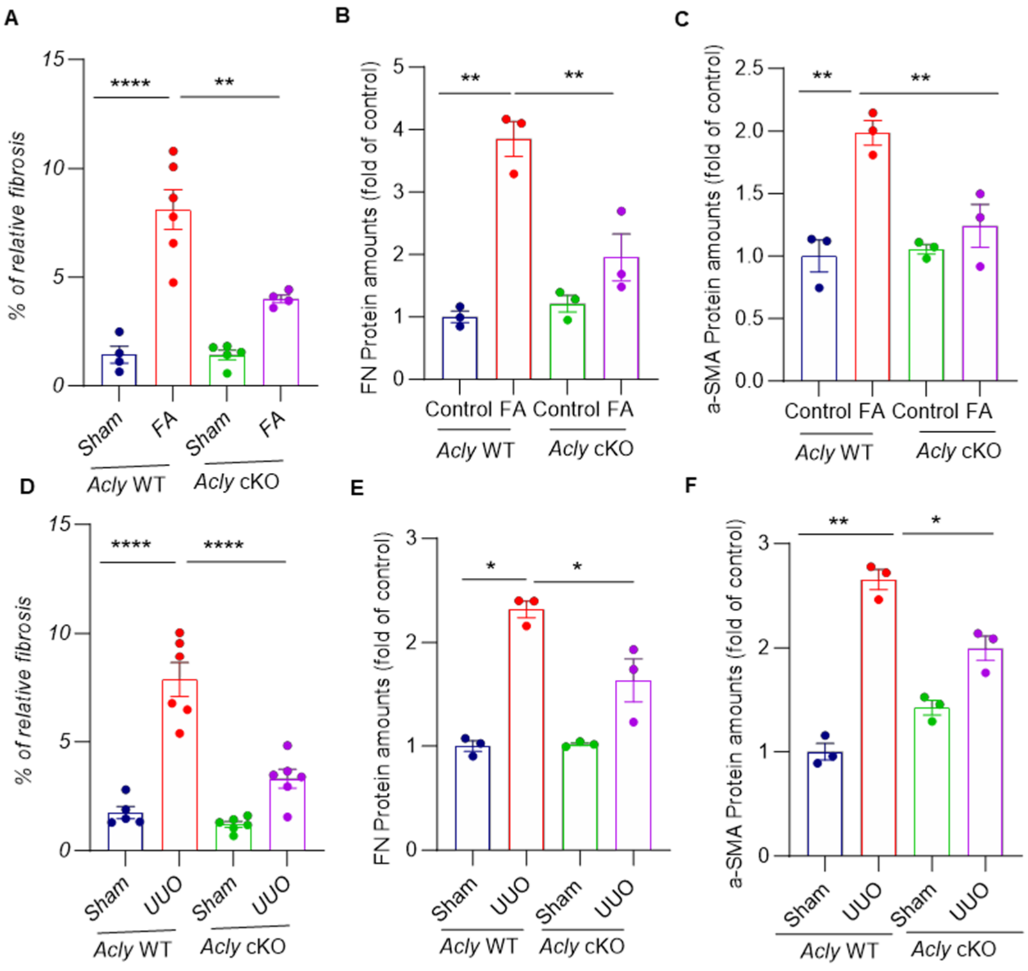

Figure S3. Quantification of fibrotic markers in *Acly* WT and *Acly* cKO mice in UUO and FA models.

(A) Quantification of Sirius Red–positive fibrosis in *Acly* WT and *Acly* cKO kidneys following FA injury. FA induced marked fibrosis in WT mice, whereas *Acly* deletion significantly reduced fibrotic deposition. n=4-6.

(B-C) Quantification of fibronectin (FN) and alpha-smooth muscle actin (α-SMA) protein levels in kidney tissues from *Acly* WT and *Acly* cKO mice in FA models. n=3.

(D) Quantification of Sirius Red–positive fibrosis in *Acly* WT and *Acly* cKO kidneys following unilateral ureteral obstruction (UUO) injury. UUO induced marked fibrosis in WT mice, whereas *Acly* deletion significantly reduced fibrotic deposition. n=5-6.

(E-F) Quantification of FN and α-SMA protein levels in kidney tissues from *Acly* WT and *Acly* cKO mice in UUO models. n=3.

Data represent mean ± SEM. Statistical analysis was performed using one-way ANOVA with Tukey's multiple comparisons test. * $P < 0.05$, **$P < 0.01$, **$P < 0.0001$.

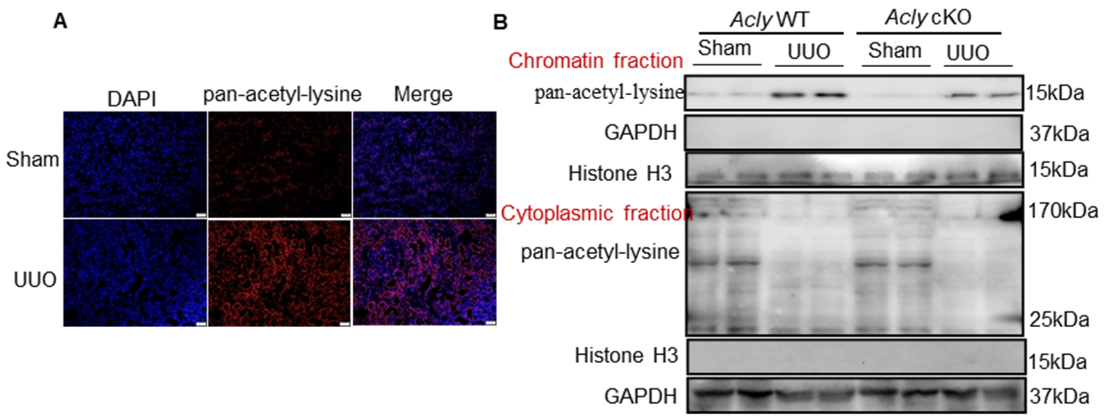

Figure S4: Global protein acetylation is markedly increased and predominantly nuclear in UUO-induced mouse models of CKD.

(A) Representative immunofluorescence images of kidney sections from control and CKD mice stained with DAPI (blue) and a pan–acetyl-lysine antibody (red). Compared with controls, CKD kidneys exhibit a robust increase in acetyl-lysine signal that shows strong spatial overlap with DAPI, indicating that global acetylation is predominantly localized within the nucleus. This nuclear enrichment is particularly evident in renal tubular cells. Merged images highlight the colocalization between acetyl-lysine staining and nuclear signals. Scale bar: 50µm.

(B) Cells were subjected to subcellular fractionation to separate chromatin-bound and cytoplasmic compartments. Pan-acetyl-lysine protein from each fraction were analyzed by immunoblotting. Fraction purity and minimal cross-contamination were verified using compartment-specific marker proteins, with GAPDH as a cytoplasmic marker and Histone H3 as a chromatin marker. Representative blots of chromatin (top) and cytoplasmic (bottom) fractions are shown.

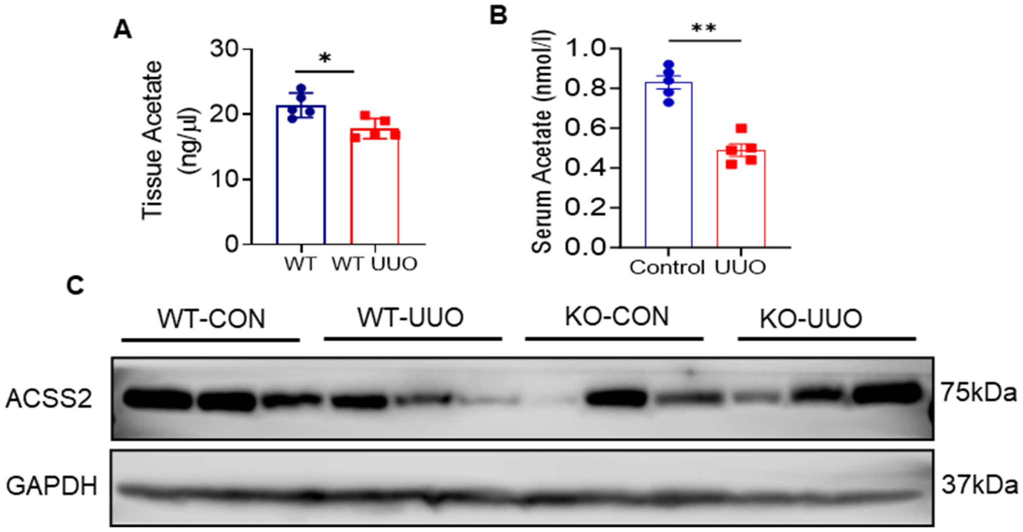

Figure S5: ACSS2 does not appear to compensate for *Acly* loss in fibrotic kidneys.
(A) Acetate levels are decreased in UUO mouse kidney tissues compared with controls.
(B) Serum acetate levels are decreased in UUO compared with Sham controls.
(C) ACSS2 protein expression in kidneys from WT-CON, WT-UUO, *Acly* cKO -CON, and *Acly* cKO-UUO mice. **P*<0.05, ***P*<0.01, n=5.

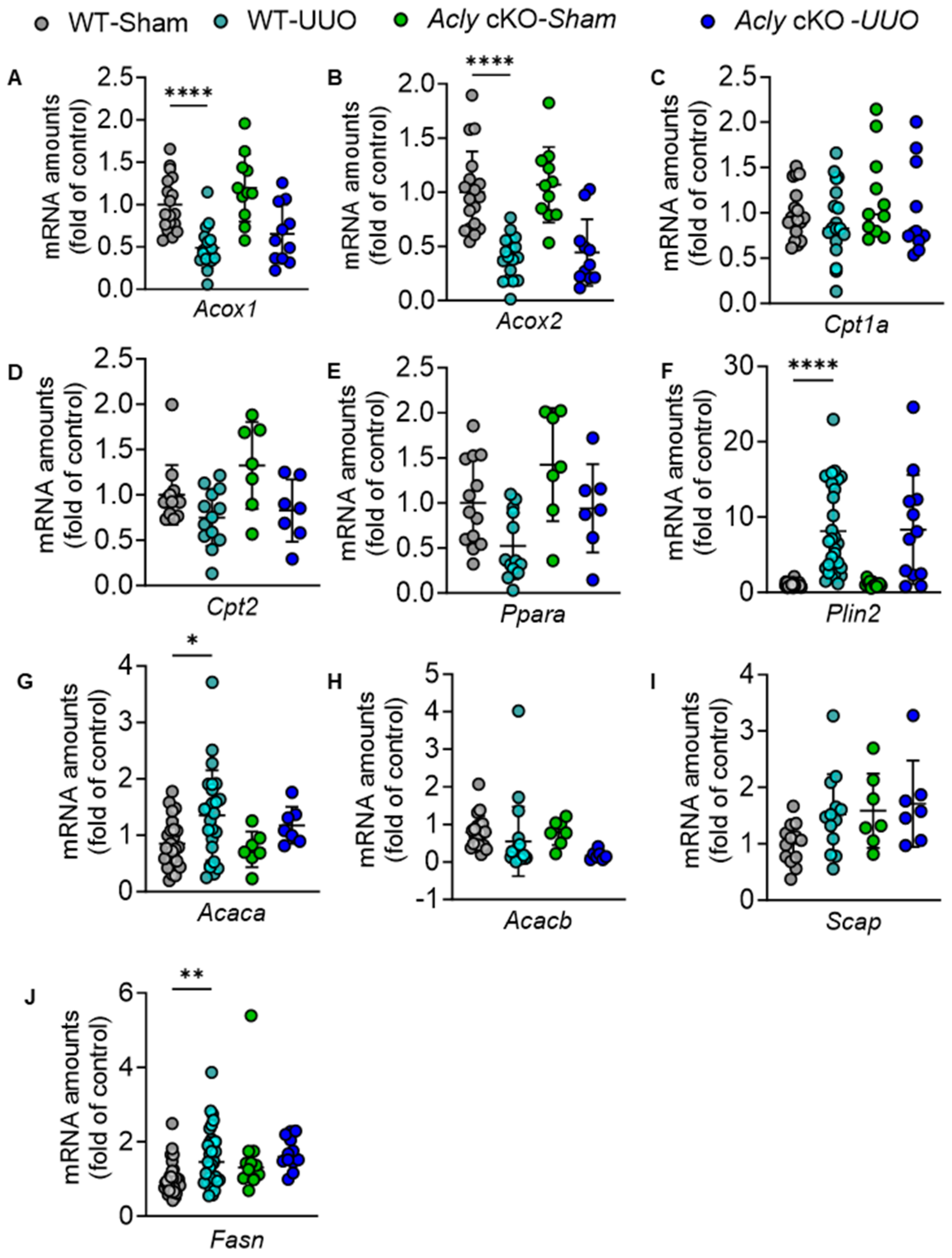

Figure S6. Altered expression of lipid metabolism–related genes in ACLY-deficient kidneys following UUO.

Quantitative RT-PCR analysis of genes involved in fatty acid oxidation and lipogenesis in kidney tissues from *Acly* WT and *Acly* cKO mice subjected to sham or UUO surgery.
(A–B) The mRNA levels of peroxisomal fatty acid oxidation genes: *Acox1* (A), *Acox2* (B).
(C–D) The mRNA levels of peroxisomal fatty acid oxidation genes of mitochondrial β-oxidation genes: *Cpt1a* (C), *Cpt2* (D).
(E–F) The mRNA levels of lipid metabolism regulators: *Ppara* (E), *Plin2* (F).
(G–H) The mRNA levels of fatty acid synthesis genes: *Acaca* (G), *Acacb* (H).
(I) The mRNA levels of lipid trafficking gene: *Scap*.
(J) The mRNA levels of lipogenesis regulators *Fasn*.
Data are shown as mean ± SEM. Statistical analysis was performed using one-way ANOVA with post hoc multiple comparisons; *$P < 0.05$, **$P < 0.01$, ****$P < 0.0001$. n=7-32.

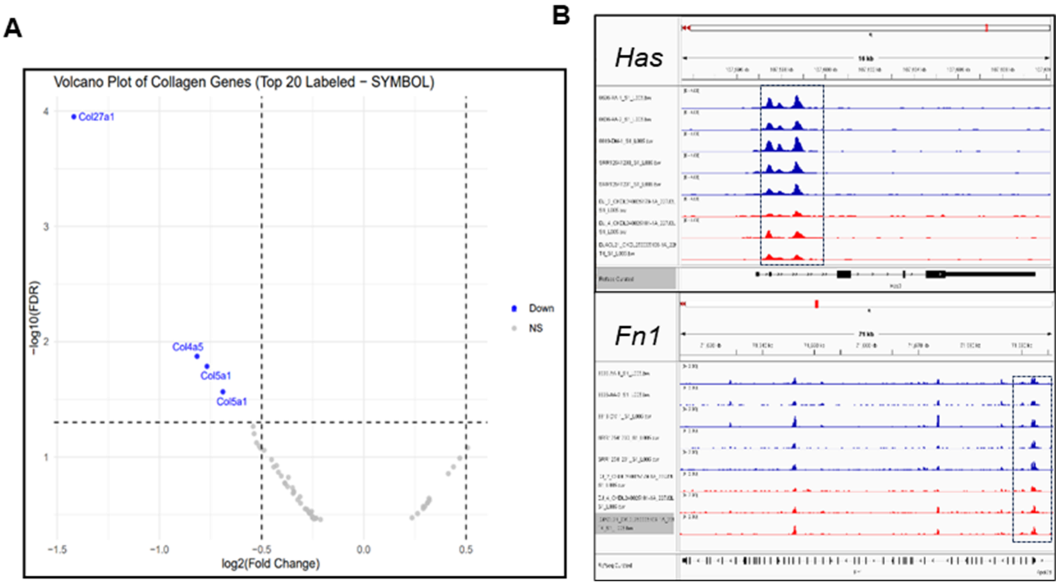

Figure S7. ***Acly* deletion reduces chromatin accessibility at profibrotic loci.** (A) volcano plot of ATAC-seq peaks corresponding to collagen genes, highlighting *Col5a1*, *Col4a5*, and *Col27a1* as among the most significantly decreased loci in *Acly* KO kidneys. (B-C) genome browser tracks showing ATAC-seq signal at representative fibrosis-associated genes, including *Has3* and *Fn1*. Compared with WT controls (blue), *Acly* KO kidneys (red) exhibit a coordinated loss of chromatin accessibility, with prominent open peaks markedly diminished at promoters and nearby enhancer regions, particularly around transcription start sites.

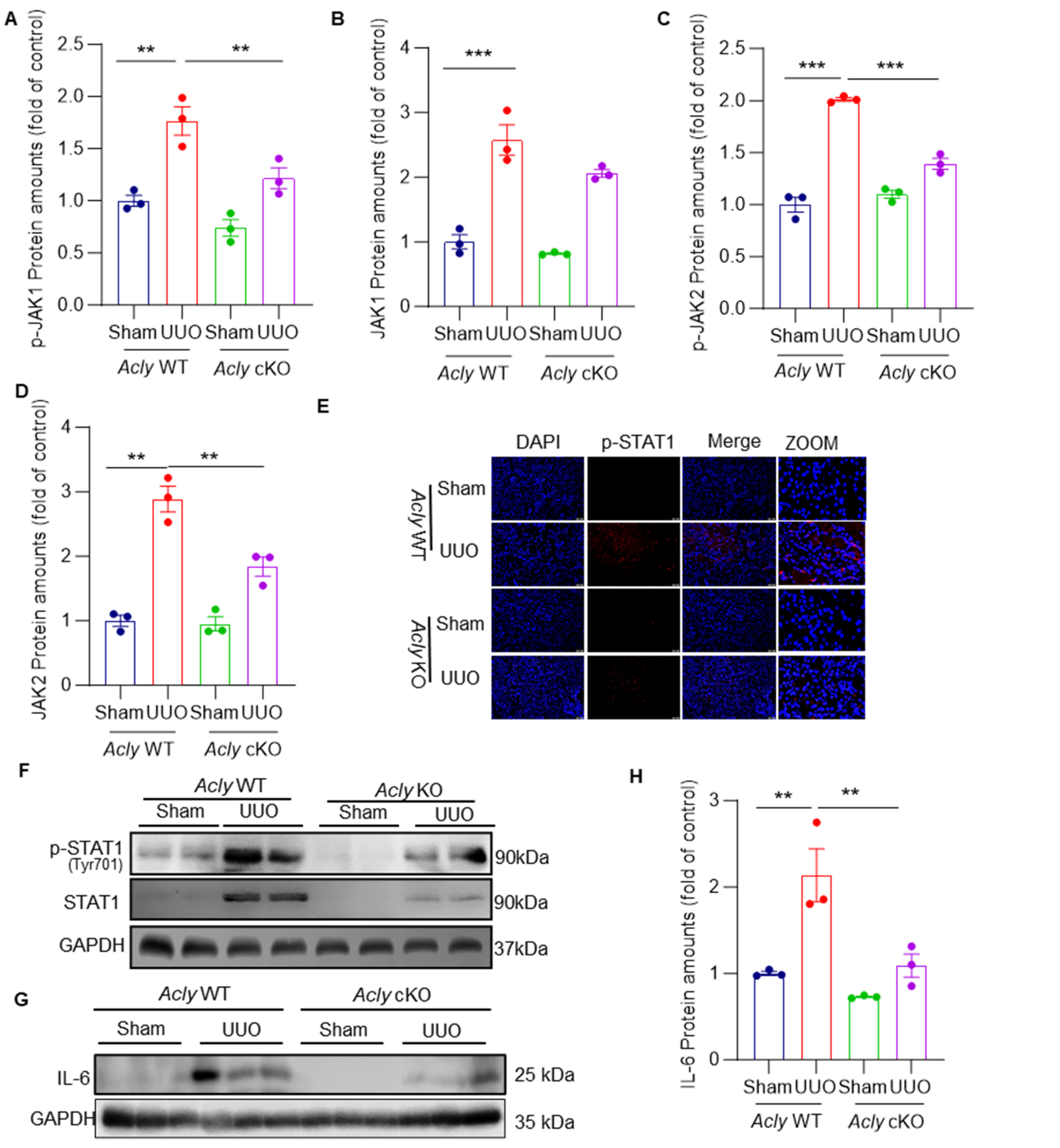

Figure S8. *Acly* deletion attenuates activation of the JAK1/2–IL-6 signaling axis in the UUO model.
(A-D) Quantification analysis of phosphorylated (A) and total JAK1 (B), phosphorylated (C) and total JAK2 (D) in kidneys from *Acly* WT and *Acly* cKO mice subjected to sham or UUO surgery.
(E) Representative immunofluorescence staining of kidney sections showing increased nuclear localization of p-STAT1(Tyr701) (red), which is attenuated by *Acly* KO. Scale bar, 50μm.
(F) Western blot analysis of kidney lysates showing increased total STAT1 and p-STAT1 (Tyr701) in UUO kidneys, which are attenuated in *Acly* KO mice.
(G-H) Western blot and quantification analysis of IL-6 protein levels in kidneys from *Acly* WT and *Acly* cKO mice subjected to sham or UUO surgery. Protein levels were normalized to GAPDH and presented as fold change relative to WT sham. Data are shown as mean ± SEM. Statistical analysis was performed using one-way ANOVA with Tukey's multiple comparisons test. $**P < 0.01$, $***P < 0.001$. n=3.

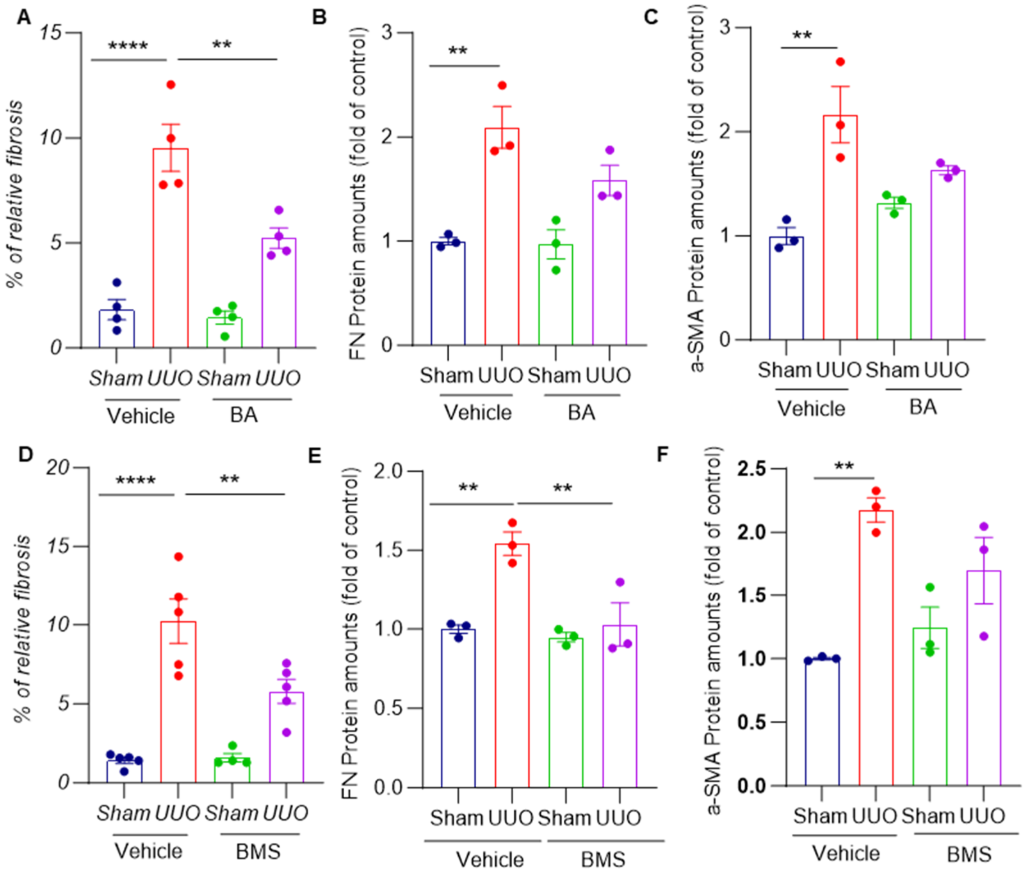

Figure S9. Quantification of fibrotic markers in WT mice treated with ACLY inhibitors in UUO models.
(A) Quantification of Sirius Red–positive fibrosis in Unilateral ureteral obstruction (UUO) kidneys treated with vehicle or bempedoic acid (BA). UUO induced robust fibrosis in vehicle-treated mice, whereas BA treatment significantly reduced fibrotic deposition. n=4.
(B-C) Quantification of fibronectin (FN) and alpha-smooth muscle actin (α-SMA) protein in kidney tissues from WT mice treated with bempedoic acid (BA) in UUO models. ***P* < 0.01. n=3.
(D) Quantification of Sirius Red–positive fibrosis in UUO kidneys treated with vehicle or BMS-303141. UUO induced marked fibrosis in vehicle-treated mice, whereas BMS-303141 treatment significantly reduced fibrotic deposition. ***P* < 0.01, *****P* < 0.0001. n=4-5.
(E-F) Quantification of FN and a-SMA protein in kidney tissues from WT mice treated with BMS303141 in UUO models. ***P* < 0.01. n=3.
Data represent mean ± SEM. Statistical analysis was performed using one-way ANOVA with Tukey’s multiple comparisons test.

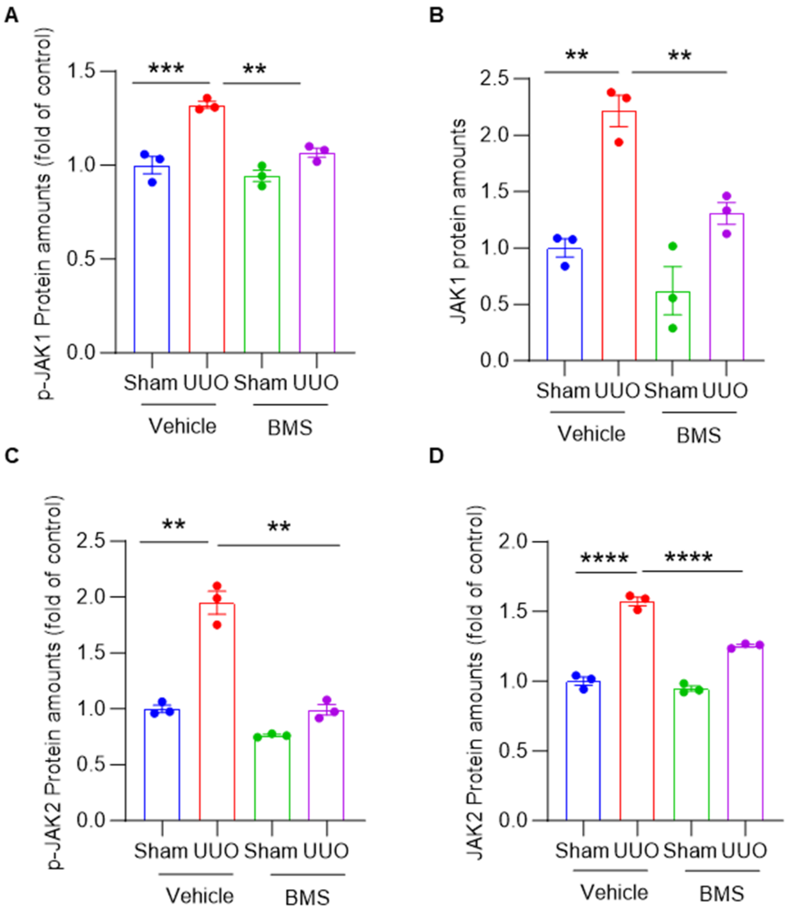

Figure S10. Quantification of JAK1/2 and phosphorylated JAK1/2 in WT mice treated with BMS 303141 in UUO models.
(A–B) Quantification of p-JAK1 and JAK1 protein in kidney tissues from WT mice treated with BMS303141 in UUO models.
(C–D) Quantification of p-JAK2 and JAK2 protein in kidney tissues from WT treated with BMS303141 in UUO models.
Data represent mean ± SEM. Unpaired two-tailed t-test for A-D. $^{**}P < 0.01$, $^{***}P < 0.001$, $^{****}P < 0.0001$. n=3.

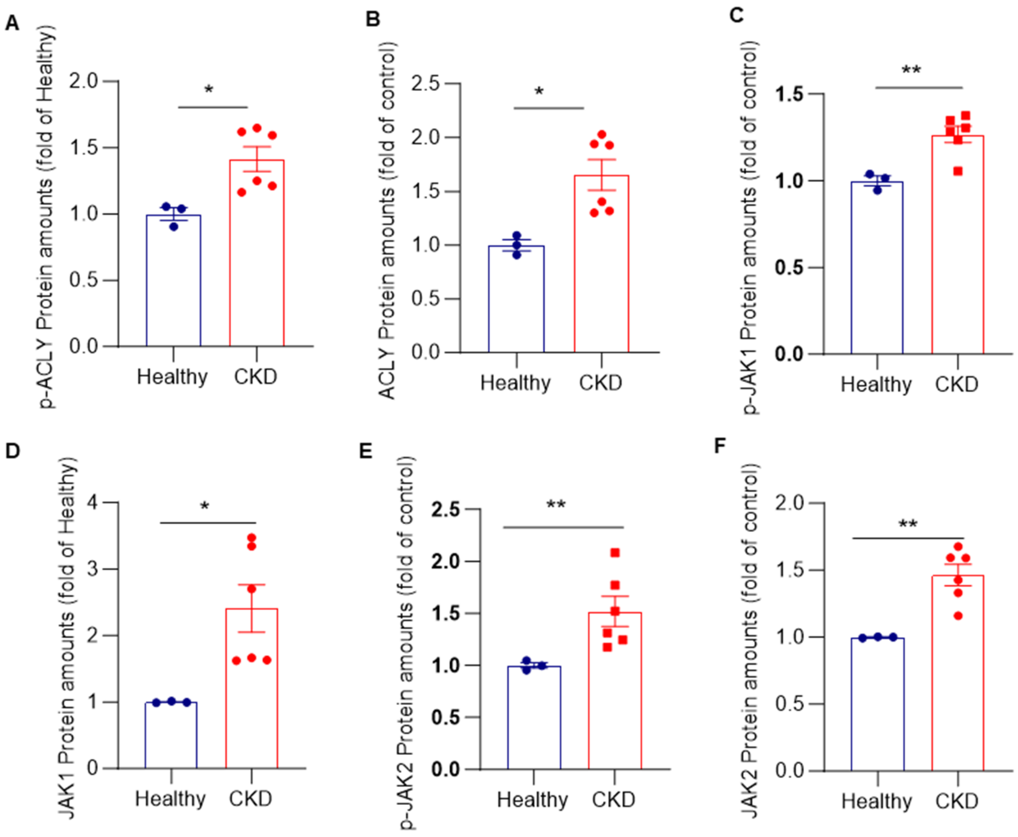

Figure S11. Upregulation of ACLY and JAK1/2 in human CKD kidneys.
Quantification of protein levels of phosphorylated ACLY (p-ACLY) (A), total ACLY (B), p-JAK1 (C), JAK1 (D), p-JAK2 (E) and JAK2 (F) in kidney tissues from healthy donors and CKD patients. Data represent mean ± SEM. Unpaired two-tailed t-test for A-F. *$P < 0.05$, ** $P < 0.01$, n=3-6
